# Priority-Setting for Novel Drug Regimens to Treat Tuberculosis: An Epidemiologic Model

**DOI:** 10.1371/journal.pmed.1002202

**Published:** 2017-01-03

**Authors:** Emily A. Kendall, Sourya Shrestha, Ted Cohen, Eric Nuermberger, Kelly E. Dooley, Lice Gonzalez-Angulo, Gavin J. Churchyard, Payam Nahid, Michael L. Rich, Cathy Bansbach, Thomas Forissier, Christian Lienhardt, David W. Dowdy

**Affiliations:** 1 Johns Hopkins University School of Medicine, Division of Infectious Diseases, Baltimore, Maryland, United States of America; 2 Johns Hopkins Bloomberg School of Public Health, Department of Epidemiology, Baltimore, Maryland, United States of America; 3 Yale School of Public Health, Department of Epidemiology of Microbial Diseases, New Haven, Connecticut, United States of America; 4 Johns Hopkins University School of Medicine, Division of Clinical Pharmacology, Baltimore, Maryland, United States of America; 5 World Health Organization, Global TB Program, Geneva, Switzerland; 6 Aurum Institute, Johannesburg, South Africa; 7 University of California San Francisco, Division of Pulmonary and Critical Care Medicine, San Francisco, California, United States of America; 8 Partners In Health, Boston, Massachusetts, United States of America; 9 Brigham and Women's Hospital, Division of Global Health Equity, Boston, Massachusetts, United States of America; 10 Bill and Melinda Gates Foundation, Seattle, Washington, United States of America; London School of Hygiene and Tropical Medicine, UNITED KINGDOM

## Abstract

**Background:**

Novel drug regimens are needed for tuberculosis (TB) treatment. New regimens aim to improve on characteristics such as duration, efficacy, and safety profile, but no single regimen is likely to be ideal in all respects. By linking these regimen characteristics to a novel regimen’s ability to reduce TB incidence and mortality, we sought to prioritize regimen characteristics from a population-level perspective.

**Methods and Findings:**

We developed a dynamic transmission model of multi-strain TB epidemics in hypothetical populations reflective of the epidemiological situations in India (primary analysis), South Africa, the Philippines, and Brazil. We modeled the introduction of various novel rifampicin-susceptible (RS) or rifampicin-resistant (RR) TB regimens that differed on six characteristics, identified in consultation with a team of global experts: (1) efficacy, (2) duration, (3) ease of adherence, (4) medical contraindications, (5) barrier to resistance, and (6) baseline prevalence of resistance to the novel regimen. We compared scale-up of these regimens to a baseline reflective of continued standard of care.

For our primary analysis situated in India, our model generated baseline TB incidence and mortality of 157 (95% uncertainty range [UR]: 113–187) and 16 (95% UR: 9–23) per 100,000 per year at the time of novel regimen introduction and RR TB incidence and mortality of 6 (95% UR: 4–10) and 0.6 (95% UR: 0.3–1.1) per 100,000 per year. An optimal RS TB regimen was projected to reduce 10-y TB incidence and mortality in the India-like scenario by 12% (95% UR: 6%–20%) and 11% (95% UR: 6%–20%), respectively, compared to current-care projections. An optimal RR TB regimen reduced RR TB incidence by an estimated 32% (95% UR: 18%–46%) and RR TB mortality by 30% (95% UR: 18%–44%). Efficacy was the greatest determinant of impact; compared to a novel regimen meeting all minimal targets only, increasing RS TB treatment efficacy from 94% to 99% reduced TB mortality by 6% (95% UR: 1%–13%, half the impact of a fully optimized regimen), and increasing the efficacy against RR TB from 76% to 94% lowered RR TB mortality by 13% (95% UR: 6%–23%). Reducing treatment duration or improving ease of adherence had smaller but still substantial impact: shortening RS TB treatment duration from 6 to 2 mo lowered TB mortality by 3% (95% UR: 1%–6%), and shortening RR TB treatment from 20 to 6 mo reduced RR TB mortality by 8% (95% UR: 4%–13%), while reducing nonadherence to the corresponding regimens by 50% reduced TB and RR TB mortality by 2% (95% UR: 1%–4%) and 6% (95% UR: 3%–10%), respectively. Limitations include sparse data on key model parameters and necessary simplifications to model structure and outcomes.

**Conclusions:**

In designing clinical trials of novel TB regimens, investigators should consider that even small changes in treatment efficacy may have considerable impact on TB-related incidence and mortality. Other regimen improvements may still have important benefits for resource allocation and outcomes such as patient quality of life.

## Abstract 2

Emily Kendall and colleagues simulate treatment regimens with differing characteristics and estimate the impacts on tuberculosis mortality and incidence.

## Author Summary

**Why Was This Study Done?:**

- Improvements in tuberculosis (TB) treatment are expected to play an important role in reaching the WHO goal of reducing TB deaths by 95%—that is, by more than 1.3 million deaths per year—between 2015 and 2035.
- Multiple aspects of existing treatment regimens have room for improvement, but it is unclear which types of improvement are most important.
- This study sought to prioritize different features of TB treatment regimens based on their potential to prevent TB deaths and new TB cases.

**What Did the Researchers Do and Find?:**

- The researchers developed a mathematical model to simulate the introduction of a novel regimen for the treatment of either drug-susceptible or multidrug-resistant (MDR) TB.
- They then compared regimens with different characteristics in terms of their ability to reduce TB deaths and new TB cases.
- Of the six characteristics modeled, regimen efficacy was the characteristic with the greatest potential to reduce TB cases and deaths, but other characteristics also had important effects (for example, shorter regimens could save health care resources).

**What Do These Findings Mean?:**

- It is important for developers of novel regimens to at least maintain the efficacy of existing regimens, and improvements in efficacy could prevent a large number of deaths.
- Other improvements, such as shorter duration and increased ease of adherence, may still have important effects by enabling more people with TB to receive appropriate and timely treatment.

## Introduction

The number of available or prospective drugs for treating tuberculosis (TB) is undergoing a long-overdue expansion. Delamanid and bedaquiline, both recently approved for the treatment of multidrug-resistant (MDR) TB [1,2], are the first novel agents registered for TB treatment in decades. Antibiotic classes such as carbapenems [3] and oxazolidinones [4] are also being repurposed to treat highly resistant TB cases. There is hope that later-generation fluoroquinolones [5], rifamycins [6], and newer drug classes [7,8] could shorten first-line treatment for TB (usually six mo), and in 2016 WHO endorsed a regimen that shortens MDR TB treatment to 9–11 mo [9] from a conventional duration of at least 18–20 mo. Despite these advances, however, many characteristics of TB regimens could be further improved, including not only treatment duration but also tolerability [10,11], efficacy [12,13], drug–drug interactions and medical indications [14,15], and the barrier against acquiring drug resistance while on therapy [16,17].

The development of improved treatment regimens within the next decade is recognized as a critical component of efforts to achieve the drastic reductions in TB cases and deaths that have been set as targets by the global community [18]. The WHO’s End TB Strategy, adopted by the World Health Assembly in 2015, highlights new drugs and shorter regimens as part of the path to a 95% reduction in global TB deaths by 2035, relative to the estimated 1.4 million that occurred in 2015 [19,20]. The Stop TB Partnership, similarly, names development of “drug regimens (including for drug-resistant TB) that are highly effective, faster-acting and nontoxic” as an essential investment if we are to meet TB elimination goals set forth in the United Nations’ Sustainable Development Goals [21]. In September 2016, WHO released target regimen profiles, describing characteristics desired in future TB regimens [22]. In the pursuit of these improved TB treatment regimens, improving all possible characteristics simultaneously in a single regimen will likely be impossible in the short term [23], leading to inevitable trade-offs. For example, higher cure rates may be difficult to achieve simultaneously with shorter treatment duration, and simpler or better-tolerated regimens may be less robust to emergence of drug resistance. Few tools currently exist to understand specific regimens’ population-level impact or to help prioritize different characteristics from this epidemiologic perspective when constructing and evaluating new regimens. We therefore developed a population-level model of novel regimens for TB, implemented within a representative set of hypothetical TB epidemics, for purposes of systematically understanding the relationships between regimen characteristics and potential population-level impact.

## Methods

**Fig 1:**
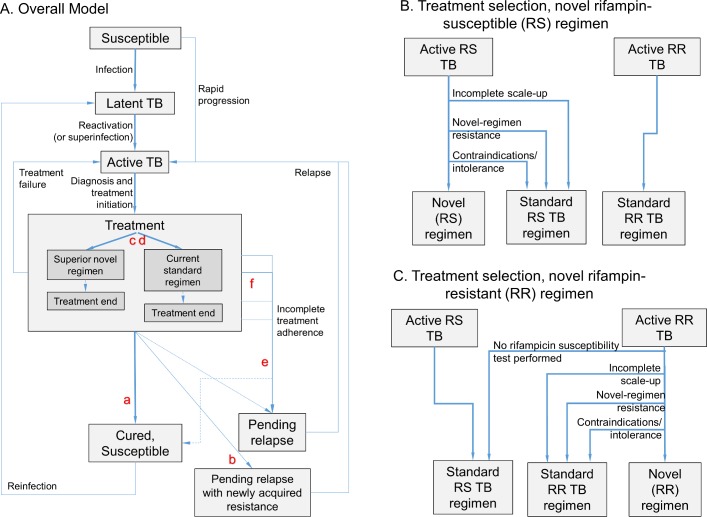
Model structure. The model (panel A) includes infection, rapid or slow progression to active TB, and initiation of treatment with a standard regimen or novel regimen (the transition from Active TB to Treatment, shown in more detail in panels B and C). (Also included in model but not shown in Fig 1: parallel structure for eight different drug resistance phenotypes; parallel structure for HIV infected/uninfected and treatment naïve/experienced; and death/spontaneous resolution.) Six novel drug regimen characteristics were evaluated within this transmission model; improved novel regimen (a) efficacy increases the probability of durable cure. A high barrier to resistance (b) prevents acquisition of resistance to drugs in the novel regimen. Less preexisting resistance to components of the novel regimen (c) and fewer medication contraindications or treatment-limiting toxicities associated with the novel regimen (d) increase the number of patients for whom the novel regimen is prescribed. Shorter regimen duration (e) and greater ease of adherence (f) both increase treatment completion, and shortened duration also reduces the probability of cure after loss to follow-up at any given time point.

We created a deterministic compartmental transmission model of a pulmonary TB epidemic in an adult population, similar to prior models with respect to the natural history of TB and HIV [24,25], but incorporating additional structure related to TB treatment and drug-susceptibility phenotypes in order to simultaneously model resistance to rifampicin and to components of novel regimens (Fig 1). Parameters related to novel regimen characteristics (Table 1) were determined through an expert consultation process described below and in S1 Methods.

**Table 1:** Modeled novel regimen characteristics and target values^*^.

Regimen characteristic	Definition of characteristic	Values modeled for novel RS TB regimen	Values modeled for novel RR TB regimen
**Efficacy**	Probability that a patient who completes the specified novel regimen duration and whose infection is and remains susceptible to the regimen will be cured without relapse**	• Minimal: 94% • Intermediate: 97% • Optimistic: 99%	• Minimal: 76% • Intermediate: 88% • Optimistic: 94%
**Barrier to resistance**	Probability that a patient treated with the novel regimen acquires and relapses with resistance to one or more components of the regimen	• Minimal: 5% • Intermediate: 0.8% • Optimistic: 0%	• Minimal: 10% • Intermediate: 5% • Optimistic: 0.8%
**Preexisting novel-regimen resistance**	Proportion of patients in the novel regimen’s targeted population (RS or RR TB) with resistance to one or more components of the novel regimen at baseline	• Minimal: 10% • Intermediate: 3% • Optimistic: 0%	• Minimal: 15% • Intermediate: 5% • Optimistic: 0%
**Medical contraindications**	Proportion of target population excluded from novel regimen treatment due to patient characteristics or adverse reactions necessitating a change of regimen***	• Minimal: 11% • Intermediate: 5% • Optimistic: 0%	• Minimal: 11% • Intermediate: 5% • Optimistic: 0%
**Duration**	Months of treatment required before the specified efficacy is achieved.	• Minimal: 6 mo • Intermediate: 4 mo • Optimistic: 2 mo	• Minimal: 20 mo • Intermediate: 9 mo • Optimistic: 6 mo
**Tolerability/ease of adherence**	Reduction in monthly nonadherence with novel regimen compared to standard regimen (due to, e.g., dosing schedule, pill burden, or route of administration)	• Minimal: 0% • Intermediate: 25% • Optimistic: 50%	• Minimal: 0% • Intermediate: 25% • Optimistic: 50%

*See S1 Methods for descriptions of the selection and estimation processes for these characteristics.

**This includes all relapses (and does not count reinfections as relapse); based on the modeled time to relapse, approximately three fourths of these relapses would be captured through 2-y follow-up.

***This parameter combined those who must receive an alternative regimen from the start and those who switch to an alternative regimen due to intolerance. We did not explicitly model impacts of side effects on quality of life and other important patient-level measures.

### TB Natural History

A complete description of the model depiction of TB natural history is provided in S2 Methods. Briefly, the risk of TB infection at each point in time reflects the number of active TB cases of each drug-susceptibility phenotype. A fraction of those who become infected (or re-infected) progress rapidly to active disease, while the remainder develop latent infection with a small but persistent hazard of reactivation. Active TB results in transmission as well as additional mortality risk, and HIV modifies multiple aspects of TB natural history.

Populations with active TB seek care and receive a TB diagnosis at a defined rate according to treatment history and HIV status. Once diagnosed, most immediately start treatment, while a smaller fraction experience pretreatment loss to follow-up and remain in the active compartment. Nonadherence is modeled as a rate of loss to follow-up each month; the modeled rate is higher than that reported in treatment cohorts, in order to account for documented losses to follow-up as well as estimates of intermittent nonadherence.

### Treatment Regimens

Three treatment regimens are modeled in each analysis: current standard of care for rifampicin-susceptible (RS) TB, standard of care for known rifampicin-resistant (RR) or MDR TB (modeled as lasting 20 mo), and a novel regimen intended for the treatment of either RS TB or RR TB. Treatment regimens are assigned on the basis of drug susceptibility testing and patient eligibility (Fig 1B and 1C), assuming gradual novel regimen scale-up over 3 y.

Novel regimens are modeled as consisting of a “companion” component (one or more drugs in current use) and a “novel” component (one or more novel agents to which resistance is negligible at baseline). Infections may be susceptible or resistant to each of the companion component, novel component, and rifampicin, for a total of 2^3^ = 8 modeled drug-susceptibility phenotypes. New resistance may be acquired during use of a regimen containing the element in question. We assume a modest 15%–45% reduction (S1 Table) in transmission fitness for infections resistant to rifampicin and/or the novel component [26,27].

We modeled introduction of a single type of novel regimen (i.e., intended either for RS TB or for RR TB) in each analysis. We assumed linear introduction of novel regimens over 3 y up to a total population coverage of 75% and measured impact at 10 y after initiation. For comparability, we assumed continued gradual scale-up of rifampicin drug-susceptibility testing (DST) through increased use of Xpert MTB/RIF or other molecular assays (as described in S2 Methods) and no other changes in current practice apart from the novel regimen, and we assumed that treatment with the novel regimen was only initiated after performing DST for drugs in the regimen.

### Treatment Outcomes

A fraction of patients treated with a given regimen are assumed to relapse with acquired drug resistance, according to a regimen’s barrier to resistance. Among other patients, the probability of durable cure reflects the fraction of the intended treatment course that is completed, the efficacy of the regimen, and the initial drug susceptibility (section 2.4 in S2 Methods). Efficacy (Table 1 and S1 Table) is defined as the proportion of patients who, in the absence of drug resistance and conditional on completing the full treatment course, experience durable cure. When durable cure is not achieved, the result may be either treatment failure (persistent active disease) or relapse to active disease after a short period of noninfectiousness (modeled as a “pending relapse” state from which relapse occurs at a specified rate).

### Model Initialization and Calibration

We started each model simulation by calibrating to epidemiologic targets based on present-day India (TB prevalence 195/100,000, HIV coprevalence 4% of individuals with TB, and RR TB 2.2% of new TB cases [28]); to explore the impact of novel regimens in epidemiologic settings with a range of TB and HIV burden, alternative analyses were also performed with the model calibrated to epidemiologic targets for Brazil, the Philippines, and South Africa (S2 Table). For each set of calibration targets, we randomly selected sets of model parameter values for a drug-susceptible TB epidemic from the ranges presented in S1 Table using Latin Hypercube Sampling (LHS). We adjusted the TB transmission rate and HIV infection rate in each simulation to achieve the target TB prevalence and HIV coprevalence when the drug-susceptible epidemic was at equilibrium. We then introduced drug resistance to each simulation by randomly sampling (again using LHS) 20 sets of parameters related to rifampicin resistance (S1 Table) for each drug-susceptible simulation—thereby resulting in 20 separate simulations for each drug-susceptible epidemic. After the introduction of rifampicin resistance, we allowed the model to progress for 25 y, reflecting the slow emergence of drug resistance over a prolonged time period prior to the historical introduction of effective second-line therapy. During the final 10 y of each calibration period, we gradually introduced second-line treatment, thereby enabling us to replicate the current situation in which most previously treated RR TB cases and a minority of treatment-naïve RR TB cases are identified and appropriately treated (S1 Table). We then evaluated the prevalence of RR TB among incident TB cases in each simulation at the end of this calibration period, excluding those that differed from our calibration target (2.2% in the primary analysis) by more than a factor of 1.5. The resulting calibrated epidemics were used to model the introduction of novel regimens at the end of this the calibration period. To ensure an adequate number of simulations, we doubled the number of simulations until results reached stability. A sensitivity analysis described in S3 Methods considers an alternative Bayesian approach to model calibration in which we weighted all simulations according to a joint Gaussian likelihood function based on WHO estimates of TB incidence, mortality, and RR TB prevalence.

### Selection of Novel Regimen Characteristics and Their Target Values

In consultation with a WHO-appointed group of experts, we selected six characteristics of novel regimens for inclusion in our model of population impact (Table 1). These characteristics were not meant to form an exhaustive list but rather were chosen based on their potential to guide drug development and their ease of conceptualization. Regimen efficacy (which refers to the proportion cured within a specified duration) was distinguished from regimen duration and from ease of adherence (defined per month of regimen duration) because of the different mechanisms by which they impact treatment effectiveness and because of the potential for tradeoffs between these characteristics. (For example, the same drug combination could be used for a shorter course with lower efficacy or for a longer course with higher efficacy, or another drug could be added to enhance efficacy and shorten duration but would reduce ease of adherence). For each characteristic, we relied on literature review and expert consultation to define a minimum acceptable value for a new regimen, an optimistic target, and an intermediate target (Table 1). S1 Methods contains additional details of the process.

For the characteristic of regimen efficacy, minimal targets for novel RS and RR TB regimens were based on the proportions achieving durable cure, among those who completed treatment, for participants in recent drug-susceptible TB treatment trials [29–31] who received standard treatment, and for patients in a systematic review of observational MDR TB cohorts [13]. Intermediate and optimistic efficacy targets represented consensus about attainable and more ambitious targets, respectively (S1 Methods 1.2.2). Targets for barrier to resistance for an RS TB regimen ranged from minimal resistance to the risk of resistance amplification for patients with isoniazid monoresistant TB treated with the standard regimen. For an RR TB regimen, this barrier ranged from that of the current standard RS TB regimen to that of current RR TB standard of care (section 1.2.3 in S1 Methods). Prevalence of preexisting resistance to the novel regimen was assumed to range from no resistance to the approximate prevalence of isoniazid and fluoroquinolone resistance among RS TB and RR TB patients, respectively (section 1.2.4 in S1 Methods). Regimen duration varied from current standard durations to the most optimistic durations considered plausible within the next decade (section 1.2.5 in S1 Methods). Proportions of patients who could be excluded from novel regimens for reasons other than drug resistance were determined by estimating the prevalence among TB patients of each of multiple possible contraindications (see list in section 1.2.6 in S1 Methods) and considering that a regimen could have zero, one, or multiple such contraindications; sensitivity analyses considered HIV-specific exclusions. Finally, the adherence characteristic combined observed rates of loss to follow-up as well as intermittent nonadherence (section 1.2.7 in S1 Methods), modeling both processes as a monthly attrition rate in order to fully capture the potential impact of shortened treatment durations on adherence and resulting effectiveness (section 2.4.2 in S2 Methods).

### Outcome Measures and Reporting

**Fig 2:**
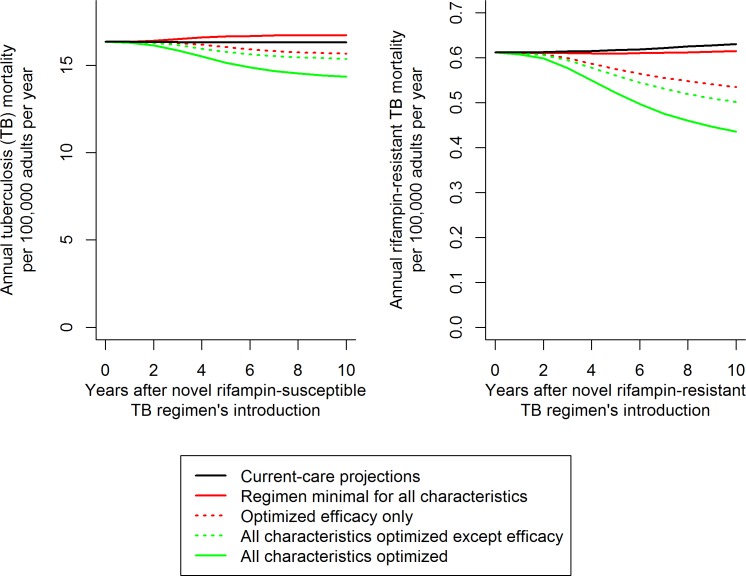
Illustration of resulting mortality trends and comparisons for different novel RS and RR TB regimens. Trajectories illustrate the median impact of novel regimens on the median projections of TB mortality. The impact of variation in each individual characteristic (such as efficacy, illustrated here) was evaluated as a fraction of the total impact of regimen optimization (distance between solid red and green trend lines). This evaluation was performed by optimizing the characteristic in question with an otherwise minimal baseline (difference between solid and dashed red lines, corresponding to the results shown in Fig 3A and 3C) and then by removing the characteristic from an otherwise optimized novel regimen (difference between solid and dashed green lines, corresponding to Fig 3B and 3D). Scale-up of the novel regimen was assumed to occur over 3 y following regimen introduction, and analyses were performed over the 10 y following the novel regimen’s introduction (including the 3 y of scale-up).

Our primary outcome was the reduction in TB mortality (for RS TB regimens) or RR TB mortality (for RR TB regimens) in the India-like setting, 10 y after introduction of a given regimen, relative to a novel regimen meeting only minimal targets and to a novel regimen meeting all optimal targets (Fig 2). Secondary outcomes included reduction in incidence, reduction in total number of patient-months on treatment, reduction in mortality in other epidemiologic settings, and reduction in mortality when regimen improvements enhanced or limited scale-up of the novel regimen (causing an RS TB regimen to reach from 50% to 100% of eligible patients after 3 y and causing an RR TB to expand its reach more quickly through accompanying accelerated scale-up of rifampicin DST).

The model was coded and statistical analyses performed in R version 3.2.3 [32]. Unless otherwise specified, results are presented as the median and 95% uncertainty range (UR) (representing the 0.025 through 0.975 quantiles) over all simulations that met calibration targets.

### Sensitivity Analyses

To understand the role of scale-up of a novel regimen, we considered variation in the mortality impact of a novel RS TB regimen as its reach ranged between 50% and 100% of eligible patients. For an RR TB regimen, we evaluated the extent to which its impact increased if it its introduction were accompanied by accelerated scale-up of rapid rifampin susceptibility testing.

We evaluated the sensitivity of the relative impact of each particular regimen characteristic, and of the total impact of a fully optimized novel regimen, to each of the model input parameters (S1 Table) by calculating partial rank correlation coefficients (PRCCs). For sensitivity analysis of the impact of HIV-specific exclusions, we modeled a scenario in which the same total fraction of patients was excluded, but those exclusions were concentrated among people living with HIV, as well as an extreme scenario in which all HIV-positive individuals in the South African setting were excluded from the novel regimen.

In structural sensitivity analyses, we tested sensitivity to our assumption of homogeneous contact structure by repeating our primary analysis after dividing the modeled population into two groups with 50% higher and 50% lower transmission rates than the base case, partitioning the population between these groups in a ratio that maintained the same overall TB prevalence. We also tested sensitivity of these impacts to our assumption of an underlying RS TB epidemic at equilibrium by instead modeling an epidemic in which TB incidence was decreasing at a rate of 2%–3%/year due to secular declines in the transmission coefficient, probability of progressing rapidly to active disease, latent TB reactivation rate, and TB diagnosis rate (the four parameters to which TB incidence was most sensitive).

## Results

### Calibration and Baseline Projections

For the evaluation of RS TB regimens in India, 4,917 simulations met calibration targets, with an estimated baseline TB incidence and mortality of 157 (95% UR: 113–187) and 16 (95% UR: 9–23) per 100,000 per year. Corresponding estimates for the 5,298 simulations calibrated to evaluate the RR TB regimen scenario were as follows: TB incidence of 143 (95% UR: 103–170), TB mortality of 16 (95% UR: 9–24), RR TB incidence of 6.0 (95% UR: 3.5–10.2), and RR TB mortality of 0.6 (95% UR: 0.3–1.1)—all expressed in units per 100,000 per year. S3 Table shows corresponding outputs for the other epidemiological settings modeled.

### Impact of an Optimal Novel Regimen

A novel regimen for RS TB, if it met all optimistic development targets (Table 1), was projected to reduce TB incidence by 12% (95% UR: 6%–22%) and TB mortality by 11% (95% UR: 6%–20%) relative to current practice at 10 y after implementation in the primary (India-like) setting. Given the much greater room for improvement in current RR TB treatment, a novel regimen for RR TB that met all optimistic targets could reduce RR TB incidence by 32% (95% UR: 18%–46%) and RR TB mortality by 30% (95% UR: 18%–44%) within 10 y.

### Primary Analysis: Relative Impact of Individual Novel Regimen Characteristics

**Fig 3:**
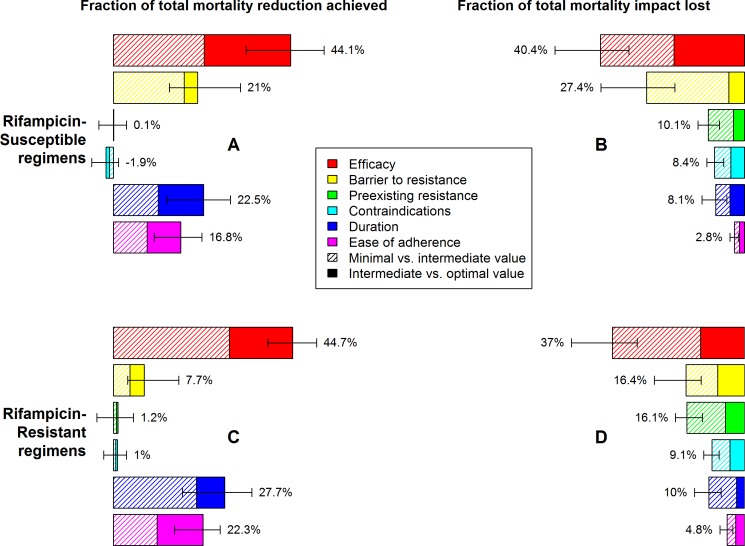
Relative mortality impact of different individual characteristics of novel regimens for the treatment of RS or RR TB. Characteristics and levels are defined in Table 1. Impact is measured as a relative change in TB mortality (RS TB regimen, A and B) or RR TB mortality (RR TB regimen, C and D) 10 y after introduction of the novel regimen, as illustrated in Fig 3. In A and C, the benefit of partially (striped bars) or fully (solid bars) optimizing only one aspect of a regimen, with the remaining characteristics meeting only minimal targets, is compared to the impact of a regimen that is fully optimized in all aspects. In B and D, the mortality reduction achievable by a regimen that fails to meet only one optimistic target (relative to mortality projections using standard regimens) is compared to mortality reduction with a regimen that meets all optimistic targets. Percentages need not sum to 100% due to synergy between multiple characteristics of the regimen. Error bars show the 95% UR for the impact of each fully optimized characteristic.

Upon varying each of the six regimen characteristics in isolation (compared to a regimen that met either minimal targets only or all optimal targets), regimen efficacy had the greatest potential impact on mortality and incidence (Fig 3 and S1 Fig). Improving the efficacy of a novel RS TB regimen from 94% (current-regimen estimate) to 99% was projected to reduce TB mortality by 6% (95% UR: 1%–13%) relative to current practice; this impact of improved efficacy alone was nearly half (44%, 95% UR: 33%–52%) of the total achievable impact of a fully optimized regimen (Fig 3A). Conversely, a novel RS TB regimen that met all other optimistic development targets except for efficacy had 60% (47%–68%) of the impact of a regimen that was fully optimized, including increased efficacy (Fig 3B). Similar results were seen for a novel RR TB regimen when efficacy was increased from 76% (estimate for current RR TB regimen) to 94% (comparable to current RS TB treatment) (Fig 3C and 3D).

The impact of shortening treatment duration on treatment outcomes and resulting TB mortality and transmission was substantial but less than that of improving regimen efficacy. Compared to a regimen with the minimal value of all characteristics, a shortening of RS TB treatment duration from 6 mo to 2 mo, or of RR TB treatment duration from 20 mo to 6 mo, achieved approximately one quarter of the mortality impact that could achieved by optimizing all six regimen characteristics rather than only the duration characteristic (Fig 3A–3C). However, this magnitude of effect was only seen in settings of poor efficacy and poor adherence; if efficacy and tolerability of the regimen were improved to optimal levels, the additional impact of achieving a short duration was limited to about 10% of total novel regimen impact (Fig 3B and 3D).

Reducing nonadherence by 50% (i.e., achieving the optimistic adherence level for a novel regimen) had similar but slightly less impact than aggressively shortening treatment duration (Fig 3) and had similarly diminishing yield as efficacy and duration improved relative to current care (Fig 3B and 3D). Among the other regimen characteristics modeled, medical contraindications and exclusions due to preexisting resistance each had negligible impact when the novel regimen offered little advantage over standard therapy (Fig 3A and 3C) but became more influential when the novel regimen was optimized in other respects (Fig 3B and 3D). Concentrating the same number of contraindications among people living with HIV (e.g., due to drug–drug interactions with antiretrovirals) had slightly greater mortality impact than other types of medical contraindications, and excluding all people living with HIV from an otherwise effective regimen would cause a very large reduction in impact in a high-HIV-prevalence setting such as South Africa (S2 Results).

Low barriers to acquired resistance could substantially reduce the impact of novel regimens. For example, even under our optimistic assumption that novel-regimen DST was available and consistently used, a low barrier to resistance (e.g., 5% of RS TB patients acquiring resistance, Fig 2A, striped yellow bar) lowered the impact of an otherwise optimal novel RS TB regimen on TB mortality by 27% (95% UR: 19–40) within this 10-y time frame. Considerations for each regimen characteristic were similar whether evaluating incidence or mortality as the outcome (S1 Fig).

### Ancillary Impact of Novel Regimen Characteristics: Resource Use and Scalability

Reductions in treatment duration, in particular, had potential ancillary effects on resource requirements. For example, reducing RS TB treatment duration from 6 to 2 mo, which we projected could reduce mortality by 22% (95% UR: 13%–29%), was also projected to reduce total patient-months of TB treatment in year 10 by 35% (95% UR: 33%–37%) (S5 Fig). By contrast, the impact of improved efficacy (and other regimen characteristics) on total treatment time reflects only the ability of such regimens to reduce the number of incident TB cases requiring treatment; thus, their treatment-related resource savings are smaller and accrue more gradually.

Although we assumed the same scale-up for all novel regimens in the primary analyses above, the potential for regimen characteristics such as improved duration or safety to facilitate wider scale-up of a novel regimen is also an important consideration. In our secondary analyses of variation in regimen scale-up, we found that the mortality impact of an optimized RS TB regimen would be twice as large if it reached all eligible patients than if it reached only half of eligible patients while the remainder continued to receive current care (14% reduction, 95% UR: 8%–26%, versus 7% reduction, 95% UR: 4%–14%). If a particular regimen characteristic, such as elimination of a cold chain requirement or the expansion of opportunities to create fixed dose combinations, allowed such a substantial increase in the proportion of eligible patients reached by a superior regimen, then that characteristic could be as influential as efficacy. However, similar to the impact of other characteristics, the impact of scalability reflected the novel regimen’s ability to offer additional advantages over standard therapy, with negligible epidemiologic advantage when a novel regimen otherwise met only minimal targets.

Similarly, if introduction of a novel regimen for treating RR TB facilitated rapid scale-up of universal rifampicin DST, the estimated impact on RR TB mortality increased: an optimized RR TB regimen could reduce RR TB mortality by 30% (95% UR: 18%–44%) under continued gradual DST scale-up, compared to 45% (95% UR: 29%–60%) when accompanied by universal RR TB detection within 3 y (S6 Fig).

### Primary Results for Other Epidemiologic Settings

Results for the other settings modeled (Brazil, the Philippines, and South Africa) were similar overall to those obtained for India (S2 Fig, S3 Fig, S4 Fig), but the high HIV coprevalence in South Africa did result in some small differences. The higher TB case fatality before people with HIV-TB coinfection start TB treatment slightly reduced the proportion of TB incidence and mortality that an optimized novel RS TB regimen could prevent (S4 Table). The higher annual infection and mortality risks in the South African setting also resulted in a small increase in the relative importance to a novel regimen’s impact of preexisting resistance and a small decrease in the relative importance of regimen duration and adherence (S4 Fig).

### Other Sensitivity Analyses

Using an “intermediate” novel regimen as the baseline for comparison, the model parameters that most influenced a standardized (meeting all intermediate targets) novel regimen’s mortality impact (S7 Fig) were the efficacy and loss to follow-up associated with the standard regimens and, for RR TB regimens, the extent of RR TB detection. The relative amounts of relapse versus failure and the timing of relapse were also important (S7 Fig).

**Fig 4:**
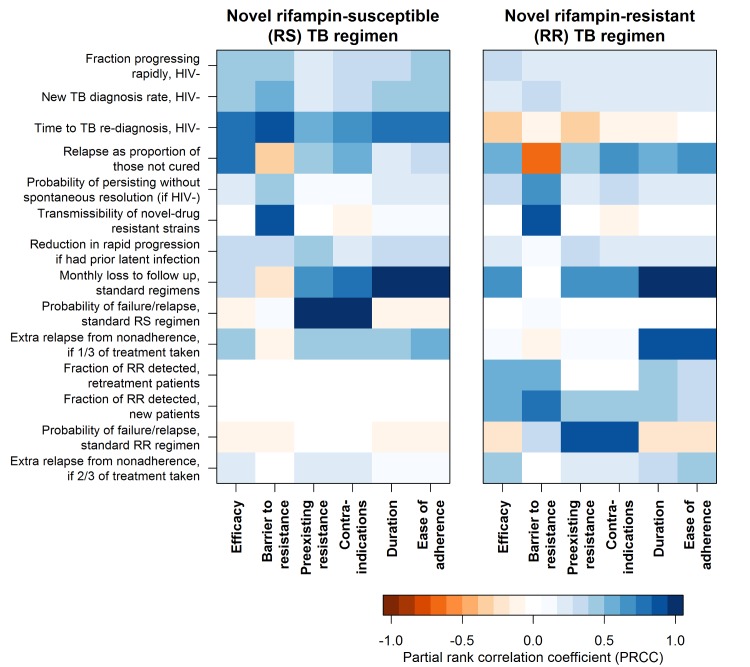
Sensitivity of the impact of individual regimen characteristics to values of model parameters. Impact of each regimen characteristic is summarized here as the difference in the percent of TB or RR TB mortality reduction that results from achieving the minimal versus the optimal target for that characteristic when intermediate targets are met for all other characteristics. For the impact of each regimen characteristic, sensitivity to model input parameters is described by the partial rank correlation coefficient, a measure of the degree of correlation between projected impact and input variable value, while holding all other input variables constant. More intense color represents greater sensitivity to the parameter, with all parameters defined such that the strongest associations are in the positive direction. Parameters that did not rank among the top four for any regimen characteristic’s impact were excluded from this figure.

The relative importance of regimen characteristics was sensitive to underlying assumptions about the values of model parameters in ways that differed between RS versus RR TB regimens (Fig 4). For example, although improvements in the efficacy of an RS TB regimen consistently had greater impact than improvements in other characteristics of an RS TB regimen, this impact was greatest when new cases were detected quickly, relapses (as opposed to outright failures, who could be immediately re-treated) were a large proportion of those not cured by treatment, and re-diagnosis of those relapses was slow. The impact of RR TB regimen efficacy improvements was instead most sensitive to the extent of RR TB detection (among both new and retreatment patients) and the amount of loss to follow-up experienced with existing regimens at baseline. Improvements in duration and ease of adherence had greater impact when rates of loss to follow-up were high at baseline and when fractional treatment courses were associated with large increases in relapse risk. Further sensitivity analysis results, including consideration of declining TB incidence and HIV-specific regimen exclusions, are shown in S2 Results, S6 Table, S7 Table, S7 Fig, S8 Fig and S9 Fig.

## Discussion

We used a dynamic transmission model in a series of idealized settings to help prioritize characteristics of novel drug regimens for treating TB. We found that increases in efficacy, for both RS TB and RR TB regimens, have the greatest potential to reduce TB incidence and mortality through direct impacts on treatment outcomes and resulting TB transmission. Shortened duration and improved tolerability may also yield substantial population-level benefits, but these will come in part through facilitating expanded treatment availability or reallocation of resources from treatment to other aspects of TB control. This process of using an epidemiological model, in ongoing consultation with worldwide experts, to help prioritize elements of new drug regimens offers a new approach to inform the development of combination antimicrobial regimens.

For RS TB regimens, our finding that further improvements in efficacy could be more important than regimen shortening runs counter to the prevailing focus on developing a non-inferior, shorter regimen. This result reflects our use of evidence that (a) existing RS TB treatment already cures a majority of patients who complete as little as 2 mo of therapy [33] and (b) 85% or more of TB patients currently complete a full course of treatment [28]. These data suggest that more patients currently relapse due to incomplete regimen efficacy rather than loss to follow-up. Unfortunately, changes of a few percentage points in efficacy may be the characteristic most difficult to demonstrate in randomized trials of feasible size and scope. This finding has important implications for clinical trial design, suggesting that non-inferiority margins for any novel RS TB regimen should be as narrow as possible to avoid unintended harm from a shorter but marginally less effective regimen. Notably, efficacy and duration of treatment are not truly independent measures; as more potent anti-TB regimens are developed, a choice may be faced between the operational benefit of reducing treatment duration and the epidemiological value of using those same potent agents for a full six mo. These results also highlight the potential importance of developing biomarkers to identify individual patients who are at highest risk for relapse and may benefit from extended or intensified therapy. For RR TB, the importance of efficacy largely reflects the poor efficacy of the existing regimen and the substantial gains that remain to be made.

Ultimately, the potential impact of novel drug regimens must be assessed from a holistic perspective; impact of specific regimen characteristics on incidence and mortality is only one consideration. In attempting to attain the ambitious targets of the End TB Strategy [34], indirect and ancillary effects of regimen improvements (for example, reduced resource requirements or improved patient experience) may be even more important, as better treatment outcomes in isolation will not achieve these goals. Specific regimen characteristics may facilitate more complete or more rapid scale-up of a more effective regimen—for instance, dosing frequency or safety monitoring requirements may determine whether a novel regimen is adopted for widespread use in particular settings. Because of unpredictability of the extent to which such features will limit uptake in different contexts, and because some such features (e.g., availability of fixed dose combinations) may be determined after a regimen is largely developed, our analysis standardized scale-up between regimens and settings. However, characteristics that determine scalability (e.g., dosing frequency or safety monitoring requirements) could be the most critical regimen characteristics in particular settings. In addition, synergies with other interventions—such as improved diagnosis, case-finding, and preventive therapy—must also be considered. For RR TB, for example, the availability of simpler and safer regimens could motivate TB programs to expand RR TB diagnosis and treatment [35], outweighing the direct effect of any particular regimen improvement in many settings. Averting adverse events such as liver, renal, and oto-toxicity is also important to individual patients, and reductions in regimen duration or visit frequency could reduce often-devastating patient costs and lost productivity [36,37].

This analysis has several limitations. First, as with all modeling analyses, we adopted a simplified structure and used parameters with substantial uncertainty. In particular, we simplified HIV natural history, age, and contact structure. These simplifications are unlikely to change the relative impact of different regimen characteristics as long as regimen improvements apply similarly across the population, but they could bias our results if simultaneous differences exist both in TB epidemiology (e.g., transmission) and in the differential impact of different regimen variables. More specific to this analysis, the particular task of linking regimen characteristics to anticipated population-level impact presents unique challenges, in that some characteristics (and the interdependence between them) are not easily represented in simplified models. In some cases, multiple regimen features all influence a single aspect of epidemic dynamics (e.g., the multiple reasons patients may be excluded from or poorly adherent to a regimen), while in other cases, a single outcome assessed during regimen development comprises multiple processes within such a mechanistic model (e.g., regimen effectiveness depends on both regimen potency and patient adherence). We therefore left the mechanism for achieving some specifications (e.g., “50% reduction in nonadherence”) open to developer interpretation. This precludes direct mapping of some elements of a typical target profile (e.g., dosing frequency or number of tablets) onto the model, while making the model better suited to its primary purpose of weighing the relative importance of different types of regimen strengths from an epidemiologic perspective. Similarly, synergies between different regimen characteristics may make it difficult to interpret a measure of an individual characteristic’s impact in the absence of a single specific regimen under study. For example, the potential gain from making a regimen more tolerable depends in part on the treatment duration, with greater impact when duration is longer (and, similarly, when efficacy is lower). The baseline and minimal levels selected may also change over time; of particular note, we used 20 mo as the worst-case duration for new RR regimens, but a 9-mo MDR TB regimen has already been endorsed for widespread use [9], setting a new benchmark for RR regimen duration and perhaps also efficacy [38]. We also deferred consideration of scalability to secondary analyses due to its context dependence, and, in doing so, we may have underestimated the impact of characteristics such as duration in those settings in which shorter duration would result in wider adoption of a novel regimen. Finally, there is much uncertainty about the selection, amplification, and transmission of drug resistance associated with novel regimens; we attempt to mitigate the effects of such uncertainty by assuming use of DST for novel regimens and by limiting analyses to a 10-y time horizon, but the relationships between preexisting drug resistance, emergence and amplification of resistance during treatment, and impact of resistance on treatment efficacy and disease transmission warrant further exploration. Consistent DST may be essential for regimens that have low barrier to resistance or significant overlap with regimens already in use.

In conclusion, this analysis suggests that TB drug development could achieve substantial impact on mortality and TB incidence by capitalizing on new, more potent TB drugs and drug combinations to improve treatment efficacy. Other regimen characteristics such as duration and safety are also critically important, but much of their impact on population-level dynamics may occur through indirect effects on the health system. The importance of even small changes in efficacy implies that the reported efficacy gains of new MDR regimens may be at least as impactful as their reduced duration [38], that clinical trials of new RS TB regimens should ensure that efficacy is at least maintained in new regimens, and that a strategy of increasing RS TB regimen potency rather than shortening duration merits further consideration. The development of novel drug regimens will be an essential component of ending the global TB epidemic, and priority-setting frameworks such as the one presented here can help to focus resources on those regimens likely to have the greatest impact at the population level.

## Supporting Information

S1 Methods Modeling of novel regimen characteristics. (DOCX)

S2 Methods Transmission model specification. (DOCX)

S3 Methods Details of model calibration and epidemiologic settings. (DOCX)

S1 Results Results of model calibration. (DOCX)

S2 Results Sensitivity of results to HIV-specific exclusions and to contact structure. (DOCX)

S1 Table Model parameters. (DOCX)

S2 Table Calibration targets for all modeled epidemiologic settings. (DOCX)

S3 Table Baseline TB incidence and mortality of calibrated models. (DOCX)

S4 Table Estimated 10-y mortality and incidence impact of optimal novel regimen for all modeled settings. (DOCX)

S5 Table Sensitivity analysis for nonequilibrium underlying RS TB epidemic: parameters used and resulting TB incidence. (DOCX)

S6 Table Sensitivity analysis results for nonequilibrium epidemic: comparing impacts of improving a single regimen characteristic. (DOCX)

S7 Table Sensitivity analysis results for nonequilibrium epidemic: comparing impacts of failing to optimize a single regimen characteristic. (DOCX)

S1 Fig Results for incidence outcome, primary (India) setting. In contrast to other figures showing impact on the TB or RR TB mortality reduction resulting from a regimen, this analysis considers the impact of different novel regimen characteristics on the regimen’s ability to reduce TB incidence (RS TB regimens) or RR TB incidence (RR TB regimens).
(TIF)

S2 Fig Impact of novel regimens on TB mortality in an epidemic reflective of Brazil (lower TB prevalence, higher HIV coprevalence). (TIF)

S3 Fig Impact of novel regimens on TB mortality in an epidemic reflective of the Philippines (high TB prevalence, low HIV coprevalence). (TIF)

S4 Fig Impact of novel regimens on TB mortality in an epidemic reflective of South Africa (high TB prevalence and high HIV coprevalence). (TIF)

S5 Fig Total patient-months on treatment resulting from different RS TB regimen improvements. Different improvements in a novel RS TB regimen have different population-level impacts on total TB treatment person-time. Efficacy improvements reduce the use of all regimens by lowering incidence most dramatically. Inclusive eligibility allows more patients to receive the novel rather than standard regimen, which reduces total treatment time only if the novel regimen is also shorter. Shortening the regimen duration has a direct and immediate impact on the total patient-months on treatment.
(TIF)

S6 Fig Role of expanded RR TB detection and treatment in novel RR TB regimen impact. This sensitivity analysis considers a scenario in which an improved RR TB regimen allows or motivates more rapid scale-up of rifampin DST, such that universal rifampin DST is achieved by the end of the 3-y scale-up period for the novel regimen. Compared to the baseline scenario that assumes continued gradual scale-up of DST, the indirect effect of simultaneous rapid DST scale-up is expected to approximately double the direct effect of a novel regimen improvement such as shortened duration or improved tolerability.
(TIF)

S7 Fig Sensitivity analysis: influence of individual parameter estimates on overall novel regimen impact. Partial rank correlation (adjusted for other parameters) was calculated for each parameter with the percent reduction in TB mortality (or, for RR TB regimens, the percent reduction in RR TB mortality) achieved by a novel regimen that met all intermediate target criteria.
(TIF)

S8 Fig Primary results with contact structure changed to heterogeneous, as described in S3 Methods. (TIF)

S9 Fig Sensitivity analysis for calibration method. The calibration method used in the primary analysis, in which all simulations that fell inside of uncertainty intervals were included in the analysis with equal weight, is compared to an alternative approach weighted according to a Gaussian-based likelihood function as described in S3 Methods. In order to summarize many results in a single figure, regimen characteristics not being varied are set at an intermediate baseline; scalability is included among the characteristics varied; and reduction in mortality is shown relative to projections without any novel regimen.
(TIF)

## Abbreviations

DST: drug-susceptibility testing
LHS: Latin Hypercube Sampling
MDR: multidrug-resistant
PRCCs: partial rank correlation coefficients
RR: rifampicin-resistant
RS: rifampicin-susceptible
TB: tuberculosis
UR: uncertainty range
