# Supplementary material for: Priority-Setting for Novel Drug Regimens to Treat Tuberculosis: An Epidemiologic Model
Source: PLoS Med. 2017 Jan 3;14(1):e1002202. doi: 10.1371/journal.pmed.1002202 (PMC5207633; doi:10.1371/journal.pmed.1002202)
Supplement: S1 Methods — (DOCX) [file pmed.1002202.s001.docx]

***Priority-setting for novel drug regimens to treat tuberculosis: An epidemiologic model***

**S1 Methods: Modeling of novel regimen characteristics**

**1.1 Selection process for novel regimen characteristics**

The aim of this process was to generate a short list of key regimen characteristics that were of relevance to developers and policymakers but also could be represented sensibly in a simple mathematical model. We initiated this process by first holding a series of discussions between an academic modeling team and individual high-level representatives from the World Health Organization and the Bill and Melinda Gates Foundation. The academic team first generated a large list of potential regimen characteristics, including possible model representations of those characteristics (i.e., parameters in the model that could be linked to each characteristic) and high/intermediate/low values for each characteristic (based on a thorough review of the available literature). Through iterative discussions within the academic team, a proposal was generated that included: (a) a shorter list of characteristics (including proposed model representations of those characteristics, proposed high/intermediate/low values, and references/explanations for each choice), (b) a model structure, and (c) primary outcomes to be generated by the model. This proposal was refined first through discussion with representatives from the World Health Organization and Bill and Melinda Gates Foundation. Following this discussion, the refined proposal was delivered in-person to a high-level representation of approximately 12 high-level stakeholders, including the leaders of the task forces delegated to develop three target regimen profiles. By the time of this meeting, preliminary sham results were also developed, in order to illustrate the potential utility of the model to inform the decision-making process. Comments from that meeting were then incorporated into a full set of draft results that were made available to an even further expanded group of stakeholders, both in electronic format (as a PowerPoint presentation) and in-person at a follow-up meeting. These stakeholders were again given the opportunity to comment before performing the final model runs and generating the initial draft manuscript. In summary, the selection process was initiated by a small academic team with early high-level buy-in and increasing involvement of a body of stakeholders, many of whom were given at least three separate opportunities (including two in-person) to provide feedback and shape the direction of the model results and communication thereof.

**1.2 Determination of target levels for novel regimen characteristics**

**1.2.1 Overall considerations**

Minimal, intermediate, and optimal levels for each characteristic were selected with the goal that the minimal target would represent the worst-case level that would allow regimen development to proceed, and the optimal target would represent the best that could be hoped for from regimens in the foreseeable future. The characteristics selected do not precisely match those expected to appear in a target regimen profile. In order to limit modeled analyses to a reasonable number of characteristics, multiple aspects of a regimen were in some cases grouped into a single modeled characteristic. For example, although multiple factors affecting eligibility of patients with other health conditions may be considered separately during drug development (for example, nephrotoxicity, teratogenicity, and cytochrome P450 interactions, to name a few), these exert epidemiologic impact through similar mechanisms by excluding a fraction of otherwise-eligible patients from receiving a novel regimen, and therefore we chose to group all medical contraindications into a single modeled novel regimen characteristic. In addition, although we recognized that some characteristics are inter-related (e.g. shortening the duration for which any particular combination of drugs is prescribed will reduce the proportion of patients cured, and sterilizing activity may be associated with both higher efficacy and lower risk of acquired drug resistance), but we model them individually in order to allow us to explore their relative impacts.

**1.2.2 Efficacy**

Novel regimen efficacy targets are defined in relation to existing-regimen benchmarks (See S2 Methods for details of the estimation of existing regimens’ efficacy). Minimal targets for novel RS-TB and RR-TB regimens (94% and 76%, respectively) are the estimated efficacy of standard RS-TB and RR-TB regimens as defined above. For RS-TB regimens, intermediate (97%) and optimal (99%) targets represent consensus opinion on possible (albeit difficult to demonstrate) improvements. For RR-TB regimens, intermediate (88%) and optimal (94%) targets reflect results of the “Bangladesh regimen” [1] and RS-TB regimen, respectively.

Note that this parameter reflects treatment outcomes for those patients who complete treatment and who are and remain susceptible to the treatment regimen. Outcomes for those who discontinue treatment and who have or acquire drug resistance are defined by other parameters below.

**1.2.3 Barrier to emergence of drug resistance**:

As described in Methods, we represent novel regimens as consisting of (1) a novel drug component with no resistance in the population at baseline but to which resistance may be acquired, and (2) one or more “companion drugs” to which some resistance may already exist.

For novel RS-TB regimens, the estimated rate at which additional drug resistance emerges in isoniazid mono-resistant patients during treatment with the standard RS-TB regimen [2,3] is taken as the worst-case barrier to new novel-drug resistance. Estimates of acquired resistance, to rifampicin or any drug in the treatment regimen, for pan-susceptible patients treated with the standard RS-TB regimen [2,4,5] are used to define an intermediate target. The optimal target reflects no acquired resistance when baseline resistance is excluded and the regimen is correctly administered.

For novel RR-TB regimens, the minimal target (worst-case barrier to resistance that might allow a novel regimen to be considered) is benchmarked to reported acquired resistance to fluoroquinolones or aminoglycosides under current standard of care [6]. The intermediate and optimal targets are set equal to the minimal and intermediate targets for an RS-TB regimen above, respectively.

Those who have preexisting or acquired resistance to one component of the novel regimen also have an increased probability of developing resistance to the other component, but under the DST assumptions of the present analysis this has minimal influence on outcomes.

**1.2.4 Prevalence of pre-existing novel-regimen (companion drug) resistance at time zero:**

For RS-TB regimens, minimal (10%) and intermediate (3%) targets for baseline resistance prevalence are benchmarked to the global prevalence of isoniazid resistance [7,8] and rifampicin resistance [9], while the optimal target is no pre-existing resistance.

For RR-TB regimens, somewhat higher minimal and intermediate targets (15% and 5%) are used to reflect the prevalence of resistance of drugs such as fluoroquinolones and pyrazinamide among RR-TB patients in some settings [10–13]. Again, the optimal target is set as no pre-existing resistance.

Note that in the analyses presented here, we assume use of DST for novel and companion components of the novel regimen, so individuals with pre-existing resistance are excluded from treatment with the novel regimen. Without the DST assumption, individuals with novel-regimen resistance may be treated with the novel regimen, and in such patients the regimen has lower efficacy and higher risk of additional acquired resistance.

**1.2.5 Duration of regimen required to be completed before full efficacy (#1 above) is achieved**:

Although for any given combination of drugs there will be a tradeoff between efficacy (when defined as prevention of relapse) and duration, we model the impact of varying only one of these parameters at a time (e.g. as might hypothetically be achieved by using different combinations or doses of drugs) in order to understand the relative importance of duration versus efficacy.

For RS-TB regimens, we use the current duration (6 months) as the minimal target, the 4 month duration evaluated in several recent and ongoing clinical trials as the intermediate target, and 2 months as the shortest duration being seriously considered for TB regimens in the near future and therefore the optimal target [14].

For RR-TB regimens, we use current duration as the minimal target (20 months), durations being evaluated for new multidrug-resistant TB regimens as the intermediate target of 9 months [15], and the duration of current standard RS-TB therapy as the optimal target.

**1.2.6 Proportion of population excluded from treatment due to medical/demographic/safety contraindications**

A variety of special populations could potentially be excluded from eligibility for a novel regimen, for example due to age, cardiac/liver/renal disease, pregnancy, interactions with essential medications such as antiretrovirals etc. Adverse reactions to the regimen could also force patients to switch to an alternative regimen shortly after starting treatment. All of these factors exert population-level impact via the same mechanism, and therefore, we modeled them collectively as a single characteristic, “Medical contraindications”.

For both RS-TB and RR-TB novel regimen, we consider the impact of such contraindications in 11% of TB patients (i.e., two of the larger and one of the smaller contraindications in the list below, although that list is not comprehensive of all possible contraindications) as a minimal breadth of eligibility, 5% as an intermediate target, and 0% (universal eligibility) as an optimal target. Examples of contraindications that could comprise these totals include:

- **Renal disease:** ~5% of patients excluded if contraindicated in chronic kidney disease stage >= 3 [16]
- **Cardiac conduction disease:** ~5% of patients excluded if contraindicated for baseline QTc interval >450 milliseconds [17,18]
- **Liver disease:** ~1-10% of patients excluded for abnormal liver function tests depending on stringency of cutoff [19,20]
- **Pregnancy:** ~1% of patients excluded due to pregnancy during a six-month treatment course (Estimating two 9-month pregnancies per woman, 37% of global TB cases occurring in women, 1/3 of those occurring during childbearing years [9], and a 6-month TB treatment duration)

Side effects requiring change in treatment could also contribute to the total percentage modeled.

In sensitivity analyses, we also consider the impact of HIV-specific exclusions, by comparing the impact of excluding 11% of all patients, independent of HIV status, to the impact of concentrating those exclusions among individuals with HIV; that is, in the primary setting, we modeled excluding all 4% of those patients with HIV plus 7% of others, and in the setting of high HIV co-prevalence (modeled after South Africa), we excluded 18% of HIV positive patients (=11% of the total number of TB patients).

Finally, our model represents an adult population, but because pediatric TB is rarely infectious, excluding children impacts only those children’s outcomes (does not impact transmission) and can be secondarily estimated from the model. We estimate pediatric incidence from the adult force of infection [21], and we use the difference in mortality after starting treatment between new and alternative regimens to estimate the impact on pediatric mortality.

**1.2.7 Tolerability / Ease of adherence**

Here, we include factors such as pill burden, dosing frequency, and inconvenient side effects that would reduce patient adherence or increase losses to follow up. (Note that duration of treatment is modeled separately and described above). Data are lacking for estimating what changes in regimens could prompt what degree of improved adherence or reduced loss to follow up, so we do not model specific characteristics such as once vs twice daily, single-pill formulations, or long-acting injectables. Instead, we make a generous estimate of the impact that a significant improvement in tolerability could have on adherence, and we model the impact of that improvement to generate a best-case estimate of the potential impact of improved adherence.

Chronic disease literature ranging from cardiovascular disease to HIV to organ transplant suggests a potential to improve adherence by about 25% to 50% by reducing dosing frequency from multiple daily doses to once daily or using single-pill combinations (with measures of adherence that varied from study to study) [22–25]. For tuberculosis, there is little evidence relating pill numbers or dosing schedules to adherence, but as an upper bound, we consider a trial of electronic reminders and adherence-focused medical management for TB patients in China, which resulted in about a 50% reduction in nonadherence [26].

We therefore use 0%, 25%, and 50% reductions in nonadherence/loss to follow up as our minimal, intermediate, and optimal targets for both RS-TB and RR-TB novel regimens.

**References**

1. Aung KJM, Van Deun A, Declercq E, Sarker MR, Das PK, Hossain MA, et al. Successful “9-month Bangladesh regimen” for multidrug-resistant tuberculosis among over 500 consecutive patients. Int J Tuberc Lung Dis Off J Int Union Tuberc Lung Dis. 2014;18: 1180–1187. doi:10.5588/ijtld.14.0100

2. Lew W, Pai M, Oxlade O, Martin D, Menzies D. Initial drug resistance and tuberculosis treatment outcomes: systematic review and meta-analysis. Ann Intern Med. 2008;149: 123–134.

3. Menzies D, Benedetti A, Paydar A, Royce S, Madhukar P, Burman W, et al. Standardized treatment of active tuberculosis in patients with previous treatment and/or with mono-resistance to isoniazid: a systematic review and meta-analysis. PLoS Med. 2009;6: e1000150.

4. Menzies D, Benedetti A, Paydar A, Martin I, Royce S, Pai M, et al. Effect of duration and intermittency of rifampin on tuberculosis treatment outcomes: a systematic review and meta-analysis. PLoS Med. 2009;6: e1000146. doi:10.1371/journal.pmed.1000146

5. Li J, Munsiff SS, Driver CR, Sackoff J. Relapse and Acquired Rifampin Resistance in HIV-Infected Patients with Tuberculosis Treated with Rifampin- or Rifabutin-Based Regimens in New York City, 1997–2000. Clin Infect Dis. 2005;41: 83–91. doi:10.1086/430377

6. Cegielski JP, Dalton T, Yagui M, Wattanaamornkiet W, Volchenkov GV, Via LE, et al. Extensive drug resistance acquired during treatment of multidrug-resistant tuberculosis. Clin Infect Dis Off Publ Infect Dis Soc Am. 2014;59: 1049–1063. doi:10.1093/cid/ciu572

7. Jenkins HE, Zignol M, Cohen T. Quantifying the burden and trends of isoniazid resistant tuberculosis, 1994-2009. PloS One. 2011;6: e22927. doi:10.1371/journal.pone.0022927

8. Cohn DL, Bustreo F, Raviglione MC. Drug-resistant tuberculosis: review of the worldwide situation and the WHO/IUATLD Global Surveillance Project. International Union Against Tuberculosis and Lung Disease. Clin Infect Dis Off Publ Infect Dis Soc Am. 1997;24 Suppl 1: S121-130.

9. Global Tuberculosis Report 2015 [Internet]. Geneva: World Health Organization; 2015. Available: http://www.who.int/tb/publications/global_report/en/

10. Kurbatova EV, Cavanaugh JS, Dalton T, Click ES, Cegielski JP. Epidemiology of pyrazinamide-resistant tuberculosis in the United States, 1999-2009. Clin Infect Dis Off Publ Infect Dis Soc Am. 2013;57: 1081–1093. doi:10.1093/cid/cit452

11. Whitfield MG, Soeters HM, Warren RM, York T, Sampson SL, Streicher EM, et al. A Global Perspective on Pyrazinamide Resistance: Systematic Review and Meta-Analysis. PloS One. 2015;10: e0133869. doi:10.1371/journal.pone.0133869

12. Kamal SMM, Hossain A, Sultana S, Begum V, Haque N, Ahmed J, et al. Anti-tuberculosis drug resistance in Bangladesh: reflections from the first nationwide survey. Int J Tuberc Lung Dis Off J Int Union Tuberc Lung Dis. 2015;19: 151–156. doi:10.5588/ijtld.14.0200

13. Islam T, Hiatt T, Hennig C, Nishikiori N. Drug-resistant tuberculosis in the WHO Western Pacific Region. West Pac Surveill Response J WPSAR. 2014;5: 34–46. doi:10.5365/WPSAR.2014.5.4.007

14. Papineni P, Phillips P, Lu Q, Cheung YB, Nunn A, Paton N. TRUNCATE-TB: an innovative trial design for drug-sensitive tuberculosis. Int J Infect Dis. 2016;45: 404. doi:10.1016/j.ijid.2016.02.863

15. Moodley R, Godec TR, STREAM Trial Team. Short-course treatment for multidrug-resistant tuberculosis: the STREAM trials. Eur Respir Rev Off J Eur Respir Soc. 2016;25: 29–35. doi:10.1183/16000617.0080-2015

16. Jha V, Garcia-Garcia G, Iseki K, Li Z, Naicker S, Plattner B, et al. Chronic kidney disease: global dimension and perspectives. The Lancet. 2013;382: 260–272. doi:10.1016/S0140-6736(13)60687-X

17. Beinart R, Zhang Y, Lima JAC, Bluemke DA, Soliman EZ, Heckbert SR, et al. The QT Interval Is Associated With Incident Cardiovascular Events: The MESA Study. J Am Coll Cardiol. 2014;64: 2111–2119. doi:10.1016/j.jacc.2014.08.039

18. Iribarren C, Round AD, Peng JA, Lu M, Klatsky AL, Zaroff JG, et al. Short QT in a Cohort of 1.7 Million Persons: Prevalence, Correlates, and Prognosis. Ann Noninvasive Electrocardiol. 2014;19: 490–500. doi:10.1111/anec.12157

19. Radcke S, Dillon JF, Murray AL. A systematic review of the prevalence of mildly abnormal liver function tests and associated health outcomes. Eur J Gastroenterol Hepatol. 2015;27: 1–7. doi:10.1097/MEG.0000000000000233

20. McLernon DJ, Donnan PT, Ryder S, Roderick P, Sullivan FM, Rosenberg W, et al. Health outcomes following liver function testing in primary care: a retrospective cohort study. Fam Pract. 2009;26: 251–259. doi:10.1093/fampra/cmp025

21. Jenkins HE, Tolman AW, Yuen CM, Parr JB, Keshavjee S, Perez-Velez CM, et al. Incidence of multidrug-resistant tuberculosis disease in children: systematic review and global estimates. Lancet. 2014;383: 1572–1579. doi:10.1016/S0140-6736(14)60195-1

22. Coleman CI, Limone B, Sobieraj DM, Lee S, Roberts MS, Kaur R, et al. Dosing frequency and medication adherence in chronic disease. J Manag Care Pharm JMCP. 2012;18: 527–539.

23. Cohen CJ, Meyers JL, Davis KL. Association between daily antiretroviral pill burden and treatment adherence, hospitalisation risk, and other healthcare utilisation and costs in a US medicaid population with HIV. BMJ Open. 2013;3. doi:10.1136/bmjopen-2013-003028

24. Sabbatini M, Garofalo G, Borrelli S, Vitale S, Torino M, Capone D, et al. Efficacy of a reduced pill burden on therapeutic adherence to calcineurin inhibitors in renal transplant recipients: an observational study. Patient Prefer Adherence. 2014;8: 73–81. doi:10.2147/PPA.S54922

25. Balu S, Simko RJ, Quimbo RM, Cziraky MJ. Impact of fixed-dose and multi-pill combination dyslipidemia therapies on medication adherence and the economic burden of sub-optimal adherence. Curr Med Res Opin. 2009;25: 2765–2775. doi:10.1185/03007990903297741

26. Liu X, Lewis JJ, Zhang H, Lu W, Zhang S, Zheng G, et al. Effectiveness of Electronic Reminders to Improve Medication Adherence in Tuberculosis Patients: A Cluster-Randomised Trial. PLoS Med. 2015;12: e1001876. doi:10.1371/journal.pmed.1001876
