# Supplementary material for: Priority-Setting for Novel Drug Regimens to Treat Tuberculosis: An Epidemiologic Model
Source: PLoS Med. 2017 Jan 3;14(1):e1002202. doi: 10.1371/journal.pmed.1002202 (PMC5207633; doi:10.1371/journal.pmed.1002202)
Supplement: S2 Methods — (DOCX) [file pmed.1002202.s002.docx]

***Priority-setting for novel drug regimens to treat tuberculosis: An epidemiologic model***

**S2 Methods: Transmission model specification**

**2.1 Model compartments/states:**

The following general states are each represented in the model by multiple compartments:

$S$ = susceptible

$L$ = latently infected

$A$ = active TB, not currently receiving treatment

$T_{1,2,...}$= on effective treatment, in one of the N different treatment periods that comprise a full course (see below)

$X$ = on ineffective treatment

$W$ = those who are currently asymptomatic following treatment but will soon relapse with active TB

Each of these general states is subdivided on the basis of:

- $h$ = TB treatment history ($S_{n}$,$L_{n}$,$A_{n}$: no priorknownhistory of active TB; $S_{p}$,$L_{p}$,$A_{p}$: previous diagnosed and/or treated for active TB. Denoted generically by $h$ for indexing purposes.)
- $j$ = Treatment regimen (Denoted by subscript $s$ for standard RS-TB regimen, $r$ for standard RR-TB regimen, or $n$ for novel regimen, or generically by $j$.)
- $k$ = TB drug susceptibility phenotype (Denoted by subscript $0$ [pan-susceptible], $R$ [rifampicin resistant], $N$ novel-drug resistant], $C$ [companion drug resistant], $RN$, $RC$, $NC$, or $RNC$ [poly-drug resistant phenotypes], or generically by $k$.)
- $i$ = HIV infection status (Denoted by subscript “+” or “$-$“, or generically for indexing purposes by $i$.)

**2.2 Model parameters**

Parameter estimates used in the baseline model of an RS-TB and RR-TB epidemic prior to novel regimen introduction are shown in S1 Table. S1 Table also lists the variable name corresponding to each parameter in the equations below.

**2.3 Model specification using differential equations**

**To simplify representation of the model’s differential equations, it will be helpful to first define and explain a few quantities:**

**Force of infection:** For eachdrug-susceptibility phenotype$k$, active TB that is untreated ($A$, for any treatment history and HIV status) or on ineffective treatment ($X$, for any HIV status and any regimen) contributes additively to the force of infection. We also calibrate a transmission coefficient,$\beta$, to the desired TB prevalence.

Of note, novel-drug resistance is assumed to be negligible in the population at time *t=0*, but once a patient acquires resistance during novel-regimen treatment and relapses with resistant disease, that resistant strain can be transmitted. For strains resistant to only the novel drug (with or without companion-drug resistance, as this is assumed to confer no additional fitness cost), . For resistance to both rifampin and novel drug, .

Resistance to the companion-drug component of the novel regimen, to which some resistance may exist in the population at baseline, is assigned to the specified fraction of the population at the start of the implementation period (*t=0*) and then may be transmitted. We assume that companion-drug resistance confers minimal fitness cost, that it is approximately at equilibrium at *t=0*, and that the dominant pathway for acquisition of resistance to the novel regimen involves acquisition of novel-drug resistance as the first step (but companion resistance is acquired as well in a fraction of those who acquire novel drug resistance).

**HIV:** We also model HIV infection, in a simplified manner. All adults face a constant risk per unit time of HIV infection. HIV increases the background mortality rate, the TB-attributable mortality rate, the probably of rapid progression when infected with TB, the rate of TB reactivation from latency, and the rate of diagnosis of active TB, as shown in the parameter table above; it also eliminates spontaneous TB resolution of active TB, which occurs at a low rate in HIV-uninfected individuals. The HIV infection rate $\pi$is calibrated jointly with$\beta$, to achieve the desired HIV co-prevalence among individuals with active TB.

$$\Phi_{i=1}=\pi\Phi_{i=0}\text{ for all HIV-uninfected states }\Phi$$

**Deaths** arise from background and TB-specific mortality rates (both HIV-dependent), for a total mortality rate:

$$Mort(t)=\sum_{i\in(+,-)} X_{i}(t)\mu_{i}+\sum_{h,k,i} A_{hki}(t)\mu'_{i}+\sum_{j,k,i} X_{jki}(t)\mu'_{i}$$

**“Births”:** Deaths in the population are replaced by new 15 year olds; the prevalence of latent TB among these 15 year olds assumes 15 years of exposure to the current total force of infection, and is apportioned to rifampin and companion-drug susceptible or resistant phenotypes based on their relative forces of infection; because we model only 10 years after introduction of the novel regimen, and because the regimen’s novel drug component is assumed not to have been used previously, latent novel-drug resistance is not assigned among these 15 year olds. Therefore, the fraction of latently-infected 15 year olds infected with strain *k* is $l_{k}(t)=\frac{FOI_{k}(t)+FOI_{k+N}(t)}{\sum_{k'} FOI_{k'}(t)}$ for phenotypes without novel-drug resistance, or $l_{k}(t)=0$for novel-drug-resistant phenotypes *k*. The remainder of 15 year olds are TB susceptible, treatment naïve, and all 15 year olds are HIV-negative.

**Treatment duration, completion, and loss to follow up:** To allow detailed modeling of regimens of different durations, time on treatmentis modeled as a series of compartments. The transition rates between these compartments are set such that the compartments have duration $t_{1}=t_{2}=t_{3}=t_{4}=$ 1 month, $t_{5}=$ 2 months, $t_{6}=t_{7}=$ 3 months, and $t_{8}=$ 8 months. Thus, the standard first line (6-month) regimen lasts for $N_{s}=$ 5 treatment periods, the standard RR-TB (20-month) regimen last for all $N_{r}=$ 8 treatment periods, and novel regimens with durations 2 months or 4 months last for $N_{n}=$ 2 or $N_{n}=$ 4 periods, respectively. The efficacy parameter $\sigma_{j}$ determines what fraction of those who complete regimen$j$without acquiring resistance are cured; of those who do not acquire resistance but are not cured, will improve but relapse, while will fail to clinically respond to treatment. Numerical estimates of $\sigma_{j}$ are explained below in “Estimation of treatment efficacy”.

Nonadherence and losses to follow up are assumed to accumulate at a monthly rate, and patients who complete only a partial treatment course still may be cured, but they are more likely to relapse, by a factor that depends on the fraction of treatment that was completed. We calculate relative risk of relapse, relative to those who complete treatment, by assuming that losses to follow up happen at the midpoint of the treatment block in which they occur. Therefore, those who begin treatment period $m\leq N_{j}$ for regimen $j$ have a probability $1-(1-\delta_{j})^{t_{m}}$of discontinuing treatment during period $m$, and those who do so are considered to have completed a fraction $f_{j}(m)=\frac{\sum_{p=1}^{m-1} t_{p}+\frac{t_{m}}{2}}{\sum_{p=1}^{N_{j}} t_{p}}$ of the full treatment course. The numerical estimation of the resulting relative risk of relapse, $r(f_{j}(m))$, is explained below under “Relapse risk after partial treatment”.

Treatment-naïve susceptibles come from replacement of deaths and from spontaneous resolution and are lost to death or infection:

$$\frac{dS_{ni}}{dt}=\left[Mort(t)\right]e^{-15\sum_{k} FOI_{k}(t)}+\nu_{i}\sum_{\text{susceptibilities }k} \left(A_{nik}(t)\right)-\left(\mu_{i}+\sum_{k} FOI_{k}(t)\right)S_{ni}(t)$$

Previously-treated susceptibles come from spontaneous resolution or successful partial or full treatment, and are lost to death or infection.

Latent infection: Latent infection compartments grow via infection and via entry of new latently-infected 15 year olds, and shrink via reactivation and death. Latently-infected individuals may become superinfected with the same or a different strain; when this occurs, the probability or rapid progression is reduced by $\lambda$ compared to those newly infected with their first strain, and for those who are superinfected but do not progress rapidly to active disease with the new strain, either the old or the new strain becomes their single latently-infecting strain with probabilities proportional to the two strains’ relative transmission fitness (we do not model mixed infections, nor superinfection of individuals who currently have active disease).

Active disease arises from rapid progression after infection or superinfection, reactivation from latency, or relapse after complete or partial treatment, and exit from active disease occurs due to treatment initiation, spontaneous resolution, or baseline or tb-related mortality.

$$\frac{dA_{nki}}{dt}=\rho_{i}S_{ni}(t)FOI_{k}(t)+r_{i}L_{nki}+\lambda\rho_{i}FOI_{k}(t)\sum_{k'} L_{nk'i}(t)-(\chi_{ni}(1-\delta_{0})+\nu_{i}+\mu_{i}+\mu'_{i})A_{nki}(t)$$

$$\begin{matrix} \frac{dA_{pki}}{dt}=\rho_{i}S_{ni}(t)FOI_{k}(t)+r_{i}L_{pki}+\lambda\rho_{i}FOI_{k}(t)\sum_{k'} L_{pk'i}(t) \\ +(1/\tau)W_{ki}(t) \\ -(\chi_{pi}(1-\delta_{0})+\nu_{i}+\mu_{i}+\mu'_{i})A_{pki}(t) \end{matrix}$$

To select the treatment regimen, drug susceptibility testing (DST) for rifampin and/or components of a novel regimen may be performed. The regimen is selected based on regimen availability, DST results, and other patient factors affecting eligibility.

When modeling an RR-TB regimen, we represent the present-day state of RR-TB under-treatment by assuming linear scale-up of rifampin DST over the past decade until RR-TB is detected and treated in approximately 20% of new patients and 70% of retreatment patients who have RR-TB infections (Table A); these levels of DST coverage are not varied between settings. Except where we explicitly model more rapid rifampin-DST scale-up in association with scale-up of the novel RR-TB regimen, we assume that current scale up continues beyond the start of analysis, at the same linear rate for new patients as over the past decade, and at a rate for retreatment patients that reduces the remaining gap in rifampin DST by half over the next decade.

Thus, taking *t=0* as the time that the novel regimen is introduced, and taking $\gamma_{n,RR},\gamma_{p,RR}$ as defined in Table A above for the novel RR-TB regimen scenario or $\gamma_{n,RS}=\gamma_{p,RS}=1$ for the RS-TB regimen scenario, rifampin DST coverage at time t for new or previously-treated patients in scenario scen=RS or RR is :

, $DST_{p\text{,scen}}(t)=\left\{\begin{matrix} \begin{matrix} 0, & t\leq-10 \end{matrix} \\ \begin{matrix} \frac{10+t}{10}\gamma_{p\text{,scen}}, & -10<t\leq0 \end{matrix} \\ \min\left(1,\gamma_{p\text{,scen}}+\frac{t}{20}(1-\gamma_{p\text{,scen}})\right),t>0 \end{matrix}\right\}$

Those with RR-TB who do not receive rifampin DST in the novel RR-TB regimen scenario are instead given the standard RS-TB regimen.

To avoid unnecessary complexity, we assume for the analyses of a novel RS-TB regimens that rifampin DST has reached universal uptake when the novel RS-TB regimen is implemented.

The availability of the novel regimen to its target population also increases linearly over time, starting from its introduction at *t=0* andincreasing linearly to a maximum availability *A* (set to 75% in our primary analysis). Because we assume novel regimen DST use, the regimen will be available only to drug susceptibility 0 for the novel RS-TB regimen and only to susceptibility R for the RR-TB regimen scenario. Thus, the availability of the novel regimen at time *t*, to those with the regimen’s target susceptibility *k*scen­­, is

$Avail_{k,\text{scenario}}(t)=\left\{\begin{matrix} 0\text{, }t<0\text{ or }k\neq\text{ target population for the scenario} \\ \frac{At}{3}\text{, }0\leq t\leq3\text{ and }k=\text{target population} \\ A,\text{ }t>3\text{ and }k=\text{target population} \end{matrix}\right\}$ .

In addition to those TB patients in the RS-TB or RR-TB target population who are excluded based on novel regimen unavailability or novel regimen resistance, an additional fraction $\zeta_{i}$ are excluded from the novel regimen for medical reasons and must instead receive a standard regimen (this fraction is allowed to depend on HIV status in sensitivity analyses, although not in our primary analysis).

For the novel RS-TB regimen scenario, the probability Pr*( j | t,h,k,I,RS)* of choosing regimen *j* at time *t* is

$$\begin{matrix} \Pr(n|h,k,i,t,RS)=\text{ }\left\{\begin{matrix} (1-\zeta_{i})Avail_{k,RS}(t) \\ 0 \\ 0 \end{matrix}\right\}\text{if }\left\{\begin{matrix} k=0 \\ k\in(N,C,NC) \\ k\in(R,RN,RC,RNC)\text{ because DST=1 at }t>0\text{ in this scenario} \end{matrix}\right\}\text{, } \\ \Pr(s|h,k,i,t,RS)=\text{ }\left\{\begin{matrix} 1-\Pr(n) \\ 1-DST_{h,RS}(t) \end{matrix}\right\}\text{if }\left\{\begin{matrix} k\in(0,N,C,NC) \\ k\in(R,RN,RC,RNC) \end{matrix}\right\}\text{,} \\ \text{and }\Pr(r|h,k,i,t,RS)=\text{ }\left\{\begin{matrix} 0 \\ DST_{h,RS}(t) \end{matrix}\right\}\text{if }\left\{\begin{matrix} k\in(0,N,C,NC) \\ k\in(R,RN,RC,RNC) \end{matrix}\right\} \end{matrix}$$

And for the novel RR-TB regimen scenario,

$$\begin{matrix} \Pr(n|h,k,i,t,RR)=\text{ }\left\{\begin{matrix} 0 \\ DST_{h,\text{RR}}(t)(1-\zeta_{i})Avail_{k,RR}(t) \\ 0 \end{matrix}\right\}\text{if }\left\{\begin{matrix} k\in(0,N,C,NC) \\ k=R \\ k\in(RN,RC,RNC) \end{matrix}\right\}\text{, } \\ \Pr(s|h,k,i,t,RR)=\text{ }\left\{\begin{matrix} 1 \\ 1-DST_{h,\text{RR}}(t) \end{matrix}\right\}\text{if }\left\{\begin{matrix} k\in(0,N,C,NC) \\ k\in(R,RN,RC,RNC) \end{matrix}\right\}\text{,} \\ \text{and }\Pr(r|h,k,i,t,RR)=\text{ }\left\{\begin{matrix} 0 \\ DST_{h,\text{RR}}(t)\left[1-(1-\zeta_{i})Avail_{k,RR}(t)\right] \\ DST_{h,\text{RR}}(t) \end{matrix}\right\}\text{if }\left\{\begin{matrix} k\in(0,N,C,NC) \\ k=R \\ k\in(RN,RC,RNC) \end{matrix}\right\} \end{matrix}$$

A the time of diagnosis and treatment initiation, a fraction of patients acquire resistance (to rifampin with probability $\eta_{sR}$ for the standard RS-TB regimen in individuals with RS-TB, or to the novel drug alone with probability $\eta_{nN}$ and both novel and companion drugs with probability $\eta_{nNC}$ (where the sum $\eta_{nN}+\eta_{nNC}$ is the total probability of acquired novel regimen resistance defined in table 1, and the ratio is estimated as 9:1 as described below).

We will define the efficacy of the novel regimen as $\sigma_{n0}$or $\sigma_{nR}$for RS-TB and RR-TB regimens,analogous to $\sigma_{s0}\text{ (=}\sigma_{sN}=\sigma_{sC}=\sigma_{sNC})$and $\sigma_{rR}\text{ (=}\sigma_{rRN}=\sigma_{rRC}=\sigma_{rRNC})$for the efficacy of the standard regimens.

Similarly, we define the proportion who become nonadherent during each month of novel regimen treatment as $\delta_{n}$ .

The novel regimens are assumed not to contain rifampin, or at least not to result in additional selection of resistance to rifampin among rifampin-susceptible TB.

Therefore, treatment begins through diagnosis and treatment initiation for those who have active disease (unless they experience pre-treatmtent loss to follow up) or are currently failing treatment, with a different probability for each regimen *j*, and effective treatment begins in the subset of these who do not experience acquired resistance or treatment failure.

The remainder of those who start treatment either fail to respond:

…or acquire resistance and move directly to a pending-relapse state for the new strain (shown below, second line of *dW(t)/dt)*.

Once response to treatment in period 1 has occurred (and those who will fail or acquire resistance have moved elsewhere), patients proceed through treatment periods 1 through *Nj* witha risk of loss to follow up during each transition:

$$\frac{dT_{mjki}}{dt}=\frac{1}{t_{m-1}}(1-\delta_{j})^{t_{m-1}}-(\mu_{i}+1/t_{m})T_{mjki}(t)\text{, }1<m\leq N_{j}$$

And finally, those who will relapse include those who were lost to follow up and will relapse, those who completed therapy but will still relapse, and those who acquired resistance and moved to the pending-relapse state for a more resistant strain:

**2.4 Details of the estimation of specific model parameters (apart from characteristics of novel regimen detailed in S1 Methods):**

**2.4.1 Estimation of treatment efficacy:**

For the standard first-line regimen, this probability of durable cure was estimated under clinical trial conditions from the outcomes of standard-regimen control groups in recent clinical trials for drug-susceptible TB (REMox [1], RIFAQUIN [2], OFLOTUB [3]). Specifically, considering the control groups from a per-protocol perspective (i.e. those who completed therapy), estimating that 3/4 of recurrences within 24 months are due to relapse in studies that did not distinguish reinfection from relapse [4], and assuming an approximately 0.5% fraction with relapse due to acquired resistance [5] and a background mortality of 1% (50 year average life expectancy for adult subjects and a six-month treatment course), we estimate outcomes of 4% relapse and 2% treatment failure or TB-related death (i.e. deaths occur at the TB mortality rate among those who are failing treatment, for a total of 2% failure + TB-related death), among those who complete treatment without baseline or acquired drug resistance. We additionally assume (with sensitivity analysis) that the same 2:1 outcome ratio of relapses to failures or deaths applies for all regimens, among regimen-susceptible patients who complete treatment.

For rifampin-resistant TB, treatment outcome data are more limited (in particular, patients often are not followed for relapse, and few modern clinical trial data exist), but we obtained an estimate of 76% cure from published cohorts [6,7] after excluding defaults. This estimate is significantly higher than programmatic treatment success rates, but those also include significant losses to follow up [8]. The even lower efficacy of the standard RS-TB regimen when used inappropriately to treat RR-TB was also estimated from available literature [9–11].

In this analysis, for simplicity, we assume perfect novel regimen DST use, so the reduced efficacy of the novel regimen when novel or companion drug resistance is present does not enter into these analyses, nor does the increased probability of selecting novel drug resistance when companion-drug resistance is present.

**2.4.2 Modeling of treatment nonadherence:**

Limited data on the timing of default for RS-TB [12] and RR-TB [13–15], suggest a constant rate of loss to follow up of approximately 1% per month throughout the treatment course (after a substantial fraction experience pre-treatment loss to follow up [16]). Similar rates are observed for both RS-TB [8] and RR-TB [6–8,17]. We attempted to also account for patients who remain on treatment but are intermittently non-adherent. One clinical trial of adherence-promoting interventions [18] found that that TB patients were less than 80% adherent during 30% of patient-months and that most of the insufficiently-adherent months occurred later in the treatment course. Estimating that adherence averages 50-60% during these insufficiently-adherent months, that the efficacy of a treatment course with incomplete adherence is a function of the total doses taken, and that nonadherence is unevenly distributed in the population, we model the combination of losses to follow up and intermittent nonadherence as a combined effective loss to follow up rate of approximately 3% per month, independent of total planned treatment duration.

**2.4.3 Estimation of relapse risk after completing a partial treatment course:**

We estimate the increase in relapse for discontinuation after 1/3 and after 2/3 of the planned course from historical outcomes for clinical trials using 2 months [19] and 4 months [20] of regimens similar to the current standard six-month regimen (but using streptomycin rather than ethambutol). Specifically, stopping a regimen of isoniazid, rifampin, streptomycin, and pyrazinamide after 2 months for intermittently-smear-positive patients resulted in 32% relapse, or 7.5x more relapse than the 4% that we estimate for the fraction relapsing after 6 months of the standard regimen under optimal conditions. Likewise, in multiple trials for smear-positive patients, stopping after 4 months caused 12% (i.e. a ~3x increase in) relapse. We assume that all who receive no treatment will relapse (except those with spontaneous resolution), and we interpolate segmental linear decreases in these factors between 0-2 months, 2-4 months, and 4-6 months – or, for regimens of durations other than six months, between 0 to 1/3, 1/3 to 2/3, and 2/3 to entirety of treatment course completed. Because the correlation between nonadherence and acquired resistance will vary between regimens, and because we vary only one regimen characteristic at a time in our primary analysis, we do not model adherence-related changes in the risk of acquired resistance.

**References**

1. Gillespie SH, Crook AM, McHugh TD, Mendel CM, Meredith SK, Murray SR, et al. Four-month moxifloxacin-based regimens for drug-sensitive tuberculosis. N Engl J Med. 2014;371: 1577–1587. doi:10.1056/NEJMoa1407426

2. Jindani A, Harrison TS, Nunn AJ, Phillips PPJ, Churchyard GJ, Charalambous S, et al. High-dose rifapentine with moxifloxacin for pulmonary tuberculosis. N Engl J Med. 2014;371: 1599–1608. doi:10.1056/NEJMoa1314210

3. Merle CS, Fielding K, Sow OB, Gninafon M, Lo MB, Mthiyane T, et al. A four-month gatifloxacin-containing regimen for treating tuberculosis. N Engl J Med. 2014;371: 1588–1598. doi:10.1056/NEJMoa1315817

4. Guerra-Assunção JA, Houben RMGJ, Crampin AC, Mzembe T, Mallard K, Coll F, et al. Recurrence due to relapse or reinfection with Mycobacterium tuberculosis: a whole-genome sequencing approach in a large, population-based cohort with a high HIV infection prevalence and active follow-up. J Infect Dis. 2015;211: 1154–1163. doi:10.1093/infdis/jiu574

5. Menzies D, Benedetti A, Paydar A, Martin I, Royce S, Pai M, et al. Effect of duration and intermittency of rifampin on tuberculosis treatment outcomes: a systematic review and meta-analysis. PLoS Med. 2009;6: e1000146. doi:10.1371/journal.pmed.1000146

6. Ahuja SD, Ashkin D, Avendano M, Banerjee R, Bauer M, Bayona JN, et al. Multidrug Resistant Pulmonary Tuberculosis Treatment Regimens and Patient Outcomes: An Individual Patient Data Meta-analysis of 9,153 Patients. PLoS Med. 2012;9: e1001300. doi:10.1371/journal.pmed.1001300

7. Weiss P, Chen W, Cook VJ, Johnston JC. Treatment outcomes from community-based drug resistant tuberculosis treatment programs: a systematic review and meta-analysis. BMC Infect Dis. 2014;14: 333. doi:10.1186/1471-2334-14-333

8. Global Tuberculosis Report 2015 [Internet]. Geneva: World Health Organization; 2015. Available: http://www.who.int/tb/publications/global_report/en/

9. Cox H, Kebede Y, Allamuratova S, Ismailov G, Davletmuratova Z, Byrnes G, et al. Tuberculosis recurrence and mortality after successful treatment: impact of drug resistance. PLoS Med. 2006;3: e384. doi:10.1371/journal.pmed.0030384

10. He GX, Xie YG, Wang LX, Borgdorff MW, van der Werf MJ, Fan JH, et al. Follow-up of patients with multidrug resistant tuberculosis four years after standardized first-line drug treatment. PloS One. 2010;5: e10799. doi:10.1371/journal.pone.0010799

11. Lan NTN null, Lademarco MF, Binkin NJ, Tung LB, Quy HT, Cĵ NV. A case series: initial outcome of persons with multidrug-resistant tuberculosis after treatment with the WHO standard retreatment regimen in Ho Chi Minh City, Vietnam. Int J Tuberc Lung Dis Off J Int Union Tuberc Lung Dis. 2001;5: 575–578.

12. Kruk ME, Schwalbe NR, Aguiar CA. Timing of default from tuberculosis treatment: a systematic review. Trop Med Int Health. 2008;13: 703–12.

13. Franke MF, Appleton SC, Bayona J, Arteaga F, Palacios E, Llaro K, et al. Risk factors and mortality associated with default from multidrug-resistant tuberculosis treatment. Clin Infect Dis. 2008;46: 1844–51.

14. Holtz TH, Lancaster J, Laserson KF, Wells CD, Thorpe L, Weyer K. Risk factors associated with default from multidrug-resistant tuberculosis treatment, South Africa, 1999-2001. Int J Tuberc Lung Dis. 2006;10: 649–655.

15. Kendall EA, Theron D, Franke MF, van Helden P, Victor TC, Murray MB, et al. Alcohol, hospital discharge, and socioeconomic risk factors for default from multidrug resistant tuberculosis treatment in rural South Africa: a retrospective cohort study. PloS One. 2013;8: e83480. doi:10.1371/journal.pone.0083480

16. MacPherson P, Houben RM, Glynn JR, Corbett EL, Kranzer K, MacPherson P, et al. Pre-treatment loss to follow-up in tuberculosis patients in low- and lower-middle-income countries and high-burden countries: a systematic review and meta-analysis. Bull World Health Organ. 2014;92: 126–138. doi:10.2471/BLT.13.124800

17. Johnston JC, Shahidi NC, Sadatsafavi M, Fitzgerald JM. Treatment outcomes of multidrug-resistant tuberculosis: a systematic review and meta-analysis. PLoS One. 2009;4: e6914.

18. Liu X, Lewis JJ, Zhang H, Lu W, Zhang S, Zheng G, et al. Effectiveness of Electronic Reminders to Improve Medication Adherence in Tuberculosis Patients: A Cluster-Randomised Trial. PLoS Med. 2015;12: e1001876. doi:10.1371/journal.pmed.1001876

19. Hong Kong Chest Service, Tuberculosis Research Centre Madras, and British Medical Research Council. A controlled trial of 2-month, 3-month, and 12-month regimens of chemotherapy for sputum-smear-negative pulmonary tuberculosis. Results at 60 months. Am Rev Respir Dis. 1984;130: 23–28. doi:10.1164/arrd.1984.130.1.23

20. Fox W. Whither short-course chemotherapy? Br J Dis Chest. 1981;75: 331–357. doi:10.1016/0007-0971(81)90022-X
