# Supplementary material for: Priority-Setting for Novel Drug Regimens to Treat Tuberculosis: An Epidemiologic Model
Source: PLoS Med. 2017 Jan 3;14(1):e1002202. doi: 10.1371/journal.pmed.1002202 (PMC5207633; doi:10.1371/journal.pmed.1002202)
Supplement: S3 Methods — (DOCX) [file pmed.1002202.s003.docx]

***Priority-setting for novel drug regimens to treat tuberculosis: An epidemiologic model***

**S3 Methods: Details of model calibration and epidemiologic settings**

**3.1 Model calibration targets:**

As discussed in the main text, an RS-TB-only epidemic at equilibrium was first calibrated by varying the TB transmission coefficient $\beta$ and the HIV infection rate $\pi$, to match the TB prevalence and HIV co-prevalence of each of the 4 settings shown in S2 Table. Then, after 25 years RR-TB acquisition and transmission was added, simulations were screened based on the proportion of TB incidence that was rifampin resistant, and those simulations more than a factor of 1.5 from the reported RR-TB proportion among new cases were rejected, while all others were included in the analysis with equal weight.

**3.2 Calibration method sensitivity analysis:**

In a sensitivity analysis, performed on a random subset of 10% of total simulations for the primary setting, we compared results with this calibration to results for an alternative calibration method, in which we included all simulations in the analysis but weighted them according to a likelihood function defined as follows: TB incidence, TB mortality, and RR fraction among incident TB cases were each treated as having an independent Gaussian distribution, with a mean at the reported point estimate, and with a standard deviation equal to one fourth of the width of the reported interval. These three types of data were assumed to be independent. For each simulation, a log likelihood for the simulation’s incidence, mortality, and new-infection RR prevalence, was computed as the sum of the logged densities of these three Gaussian distributions, and simulations were weighted according to the resulting likelihood in the subsequent analyses; results are provided in S9 Figure.
