# Supplementary material for: Priority-Setting for Novel Drug Regimens to Treat Tuberculosis: An Epidemiologic Model
Source: PLoS Med. 2017 Jan 3;14(1):e1002202. doi: 10.1371/journal.pmed.1002202 (PMC5207633; doi:10.1371/journal.pmed.1002202)
Supplement: S1 Results — (DOCX) [file pmed.1002202.s004.docx]

***Priority-setting for novel drug regimens to treat tuberculosis: An epidemiologic model***

**S1 Results: Calibration**

We calibrated 1,000 simulated epidemics to India’s TB and HIV prevalence, resulting in 20,000 simulated RR-TB-containing epidemics for each novel regimen scenario. Of these, 4917 simulations met targeted present-day RR-TB prevalence for the RS-TB regimen scenario (i.e. with the simplification that all RR-TB detection and RR-TB treatment scale-up reached all diagnosed TB patients by the end of the calibration period), and 5298 simulations met RR-TB prevalence targets for the novel RR-TB regimen scenario (i.e. with still-incomplete RR-TB detection at the start of analysis).

Correspondence to calibration targets for all epidemiologic settings are shown in S3 Table. 10 year projections of incidence and mortality under current care are shown in S4 Table.
