# Supplementary material for: Priority-Setting for Novel Drug Regimens to Treat Tuberculosis: An Epidemiologic Model
Source: PLoS Med. 2017 Jan 3;14(1):e1002202. doi: 10.1371/journal.pmed.1002202 (PMC5207633; doi:10.1371/journal.pmed.1002202)
Supplement: S2 Results — (DOCX) [file pmed.1002202.s005.docx]

***Priority-setting for novel drug regimens to treat tuberculosis: An epidemiologic model***

**S2 Results: Sensitivity to HIV-specific exclusions and to contact structure**

*Sensitivity to HIV-specific exclusions*

Concentrating contraindications to the novel regimen among HIV-positive patients could more substantially impact the novel regimen’s mortality impact. In the Indian (4% co-prevalence) setting, excluding all HIV-positive and 7% of HIV-negative TB patients (to comprise 10% of TB patients overall) resulted in loss of 15.7% (12.7-22.0%) of the novel regimen’s total mortality impact, compared to a loss of 10.1% (9.4-10.6%) of the novel regimen’s impact when exclusions were distributed evenly over all TB patients independent of HIV status. This was due in part to the shorter duration of TB disease in HIV (such that excluding the same proportion of prevalent cases excluded a greater number of incident cases) and in part due to the higher mortality of HIV-associated TB.

In the South African setting (where 61% of TB patients had HIV), if all people living with HIV were excluded from an otherwise-optimal novel RS-TB regimen, it would be expected to achieve only 29% (16-47%) of the reduction in TB mortality that would be expected if HIV patients were also eligible for treatment.

*Sensitivity to contact structure*

The heterogeneous contact structure we created resulted in a sub-population with TB prevalence median 8 (IQR 5-23) times higher than in the rest of the population.

With this heterogeneous contact structure, the projected reduction in TB mortality from an all-optimal novel regimen in the primary (India) setting was 10.2% (95% UR 9.5-10.6%), compared to 10.1% (9.6-10.5%) with the homogeneous contact structure. Results for the relative importance of different regimen characteristics were also similar, as shown in S8 Figure.
