# Supplementary material for: Priority-Setting for Novel Drug Regimens to Treat Tuberculosis: An Epidemiologic Model
Source: PLoS Med. 2017 Jan 3;14(1):e1002202. doi: 10.1371/journal.pmed.1002202 (PMC5207633; doi:10.1371/journal.pmed.1002202)
Supplement: S1 Table — (DOCX) [file pmed.1002202.s006.docx]

***Priority-setting for novel drug regimens to treat tuberculosis: An epidemiologic model***

**S1 Table: Model parameters.** (See main text for values of novel regimen-related parameters.)

| **Parameter** | **Median estimate (sampled range)** | **References** | **Variable used** |
| --- | --- | --- | --- |
| **Baseline mortality rate** ^(a)^ |  |  |  |
| Non-HIV | 0.012 (0.06-0.018) | [1] | __ |
| HIV | 0.033 (0.017-0.049) | [2] | $\mu_{+}$ |
| **TB-associated mortality rate** |  |  |  |
| Non-HIV | 0.1 (0.05-0.15) | [3] | $\mu'_{-}$ |
| HIV | 0.4 (0.2-0.6) | [4,5] | $\mu'_{+}$ |
| **Fraction progressing rapidly after TB infection** |  |  |  |
| Non-HIV | 0.13 (0.07-0.19) | [6] | $\rho_{-}$ |
| HIV | 0.50 (0.25-0.75) | [7] | $\rho_{+}$ |
| **TB Reactivation rate** |  |  |  |
| Non-HIV | .0015 (.0008-.0022) | [8,9] | $\alpha_{-}$ |
| HIV | .0300 (.0150-.0450) | [10] | $\alpha_{+}$ |
| **Relative probability of rapid progression, subsequent versus initial infection** | 0.5 (0.25-0.75) | [6,11] | $\lambda$ |
| **Rate of spontaneous resolution (non-HIV only)** | 0.2 (0.1-0.3) | [3,12] | $\nu_{-}$ $(\nu_{+}=0)$  |
| **Time from treatment completion to relapse** for those who will relapse (years) | 1.0 (0.67-2) ^(b)^ | [13] | $\tau$ |
| **Proportion lost to follow up** per month on treatment | 0.03 (0.015-0.045) | [14–17] | $\delta_{s}=\delta_{r}$ |
| **Relative risk of relapse risk after partial treatment course** |  | [18,19] |  |
| 1/3 of doses completed | 3.0 (1.5-4.5) |  | $\omega_{1}$ |
| 2/3 of doses completed | 7.5 (3.8-10.2) |  | $\omega_{2}$ |
| **TB diagnosis and treatment initiation rate**, per year |  |  |  |
| Non-HIV, treatment-naïve | 0.7 (0.35-1.05) | [20,21] | $\chi_{n-}$ |
| Non-HIV, previously-treated | 1 (0.5-1.5) |  | $\chi_{n+}$ |
| HIV, treatment-naïve | 2.0 (1.0-3.0) | [4,22] | $\chi_{p-}$ |
| HIV, previously-treated | 3.0 (1.5-4.5) |  | $\chi_{p+}$ |
| Patients failing current treatment | 3.0 (2.0-6.0)** |  | $\chi_{f}$ |
| **Fraction experiencing pre-treatment loss to follow up** | 0.15 (0.08-0.22) | [23] | $\xi$ |
| **Treatment efficacy of standard TB regimens** ^(c)^ |  |  |  |
| Standard rifampicin-susceptible (RS) TB regimen for RS-TB | 0.94 (0.91-0.97) | [24–26] | $\sigma_{s0}$ |
| Standard rifampicin-resistant (RR) TB regimen for RR-TB | 0.76 (0.64-0.88) | [16,27] | $\sigma_{rR}$ |
| **Fraction of non-cures ending in relapse rather than failure or TB-attributable death ^(d)^** | 0.67 (0.34-1) | [24–26] |  |
| **Probability of acquiring rifampin resistance during standard RS-TB treatment** | 0.008 | [28,29] | $\eta_{sR}$ |
| **Probability of durable cure for RR-TB patients completing standard RS-TB regimen** | 0.2 (0.1-0.3) | [30–32] | $\sigma_{sR}$ |
| **Fraction of RR-TB detected at treatment initiation ^(e)^** |  |  |  |
| New TB patients | 0.20 (0.10-0.30) | [20] | $\gamma_{n,RR}$ |
| Retreatment patients | 0.70 (0.55-0.85) |  | $\gamma_{p,RR}$ |
| **Relative transmissibility of drug-resistant strains** | 0.7 (0.55-0.85) | [33,34] |  |

(a) rates are expressed per year unless otherwise noted.

(b) Parameter sampled (from 50% to 150%) is the reciprocal of parameter listed here.

(c) Efficacy is defined as probability of durable cure for regimen-susceptible patients who complete treatment without acquiring resistance; see text for explanation of estimates.

(d) Excludes additional relapse due to nonadherence or acquired resistance.

(e) Incomplete RR-TB detection is modeled in novel RR-TB regimen scenario only; see text and appendix for details of scale-up.

**References**

1. Global Health Observatory data repository [Internet]. World Health Organization; Available: http://apps.who.int/gho/data/?theme=main&vid=61830

2. AIDS by The Numbers [Internet]. Geneva: UNAIDS; 2015. Available: http://www.unaids.org/sites/default/files/media_asset/AIDS_by_the_numbers_2015_en.pdf

3. Tiemersma EW, van der Werf MJ, Borgdorff MW, Williams BG, Nagelkerke NJD. Natural History of Tuberculosis: Duration and Fatality of Untreated Pulmonary Tuberculosis in HIV Negative Patients: A Systematic Review. PLoS One. 2011;6: e17601. doi:10.1371/journal.pone.0017601

4. Wood R, Middelkoop K, Myer L, Grant AD, Whitelaw A, Lawn SD, et al. Undiagnosed tuberculosis in a community with high HIV prevalence: implications for tuberculosis control. Am J Respir Crit Care Med. 2007;175: 87–93. doi:10.1164/rccm.200606-759OC

5. Corbett EL, Watt CJ, Walker N, et al. The growing burden of tuberculosis: Global trends and interactions with the hiv epidemic. Arch Intern Med. 2003;163: 1009–1021. doi:10.1001/archinte.163.9.1009

6. Vynnycky E, Fine PE. The natural history of tuberculosis: the implications of age-dependent risks of disease and the role of reinfection. Epidemiol Infect. 1997;119: 183–201.

7. Daley CL, Small PM, Schecter GF, Schoolnik GK, McAdam RA, Jacobs WR, et al. An outbreak of tuberculosis with accelerated progression among persons infected with the human immunodeficiency virus. An analysis using restriction-fragment-length polymorphisms. N Engl J Med. 1992;326: 231–235. doi:10.1056/NEJM199201233260404

8. Horsburgh CR, O’Donnell M, Chamblee S, Moreland JL, Johnson J, Marsh BJ, et al. Revisiting rates of reactivation tuberculosis: a population-based approach. Am J Respir Crit Care Med. 2010;182: 420–425. doi:10.1164/rccm.200909-1355OC

9. Fox GJ, Barry SE, Britton WJ, Marks GB. Contact investigation for tuberculosis: a systematic review and meta-analysis. Eur Respir J. 2013;41: 140–156. doi:10.1183/09031936.00070812

10. Antonucci G, Girardi E, Raviglione MC, Ippolito G. Risk factors for tuberculosis in HIV-infected persons. A prospective cohort study. The Gruppo Italiano di Studio Tubercolosi e AIDS (GISTA). JAMA. 1995;274: 143–148.

11. Andrews JR, Noubary F, Walensky RP, Cerda R, Losina E, Horsburgh CR. Risk of Progression to Active Tuberculosis Following Reinfection With Mycobacterium tuberculosis. Clin Infect Dis. 2012;54: 784–791. doi:10.1093/cid/cir951

12. Dye C, Garnett GP, Sleeman K, Williams BG. Prospects for worldwide tuberculosis control under the WHO DOTS strategy. The Lancet. 1998;352: 1886–1891. doi:10.1016/S0140-6736(98)03199-7

13. Marx FM, Dunbar R, Enarson DA, Williams BG, Warren RM, Spuy GD van der, et al. The Temporal Dynamics of Relapse and Reinfection Tuberculosis After Successful Treatment: A Retrospective Cohort Study. Clin Infect Dis. 2014;58: 1676–1683. doi:10.1093/cid/ciu186

14. Kruk ME, Schwalbe NR, Aguiar CA. Timing of default from tuberculosis treatment: a systematic review. Trop Med Int Health. 2008;13: 703–12.

15. Johnston JC, Shahidi NC, Sadatsafavi M, Fitzgerald JM. Treatment outcomes of multidrug-resistant tuberculosis: a systematic review and meta-analysis. PLoS One. 2009;4: e6914.

16. Ahuja SD, Ashkin D, Avendano M, Banerjee R, Bauer M, Bayona JN, et al. Multidrug Resistant Pulmonary Tuberculosis Treatment Regimens and Patient Outcomes: An Individual Patient Data Meta-analysis of 9,153 Patients. PLoS Med. 2012;9: e1001300. doi:10.1371/journal.pmed.1001300

17. Liu X, Lewis JJ, Zhang H, Lu W, Zhang S, Zheng G, et al. Effectiveness of Electronic Reminders to Improve Medication Adherence in Tuberculosis Patients: A Cluster-Randomised Trial. PLoS Med. 2015;12: e1001876. doi:10.1371/journal.pmed.1001876

18. Hong Kong Chest Service, Tuberculosis Research Centre Madras, and British Medical Research Council. A controlled trial of 2-month, 3-month, and 12-month regimens of chemotherapy for sputum-smear-negative pulmonary tuberculosis. Results at 60 months. Am Rev Respir Dis. 1984;130: 23–28. doi:10.1164/arrd.1984.130.1.23

19. Fox W. Whither short-course chemotherapy? Br J Dis Chest. 1981;75: 331–357. doi:10.1016/0007-0971(81)90022-X

20. Global Tuberculosis Report 2015 [Internet]. Geneva: World Health Organization; 2015. Available: http://www.who.int/tb/publications/global_report/en/

21. Dowdy DW, Basu S, Andrews JR. Is passive diagnosis enough? The impact of subclinical disease on diagnostic strategies for tuberculosis. Am J Respir Crit Care Med. 2013;187: 543–551. doi:10.1164/rccm.201207-1217OC

22. Corbett EL, Marston B, Churchyard GJ, De Cock KM. Tuberculosis in sub-Saharan Africa: opportunities, challenges, and change in the era of antiretroviral treatment. The Lancet. 2006;367: 926–937. doi:10.1016/S0140-6736(06)68383-9

23. MacPherson P, Houben RM, Glynn JR, Corbett EL, Kranzer K, MacPherson P, et al. Pre-treatment loss to follow-up in tuberculosis patients in low- and lower-middle-income countries and high-burden countries: a systematic review and meta-analysis. Bull World Health Organ. 2014;92: 126–138. doi:10.2471/BLT.13.124800

24. Gillespie SH, Crook AM, McHugh TD, Mendel CM, Meredith SK, Murray SR, et al. Four-month moxifloxacin-based regimens for drug-sensitive tuberculosis. N Engl J Med. 2014;371: 1577–1587. doi:10.1056/NEJMoa1407426

25. Merle CS, Fielding K, Sow OB, Gninafon M, Lo MB, Mthiyane T, et al. A four-month gatifloxacin-containing regimen for treating tuberculosis. N Engl J Med. 2014;371: 1588–1598. doi:10.1056/NEJMoa1315817

26. Jindani A, Harrison TS, Nunn AJ, Phillips PPJ, Churchyard GJ, Charalambous S, et al. High-dose rifapentine with moxifloxacin for pulmonary tuberculosis. N Engl J Med. 2014;371: 1599–1608. doi:10.1056/NEJMoa1314210

27. Weiss P, Chen W, Cook VJ, Johnston JC. Treatment outcomes from community-based drug resistant tuberculosis treatment programs: a systematic review and meta-analysis. BMC Infect Dis. 2014;14: 333. doi:10.1186/1471-2334-14-333

28. Menzies D, Benedetti A, Paydar A, Martin I, Royce S, Pai M, et al. Effect of duration and intermittency of rifampin on tuberculosis treatment outcomes: a systematic review and meta-analysis. PLoS Med. 2009;6: e1000146. doi:10.1371/journal.pmed.1000146

29. Li J, Munsiff SS, Driver CR, Sackoff J. Relapse and Acquired Rifampin Resistance in HIV-Infected Patients with Tuberculosis Treated with Rifampin- or Rifabutin-Based Regimens in New York City, 1997–2000. Clin Infect Dis. 2005;41: 83–91. doi:10.1086/430377

30. Cox H, Kebede Y, Allamuratova S, Ismailov G, Davletmuratova Z, Byrnes G, et al. Tuberculosis recurrence and mortality after successful treatment: impact of drug resistance. PLoS Med. 2006;3: e384. doi:10.1371/journal.pmed.0030384

31. He GX, Xie YG, Wang LX, Borgdorff MW, van der Werf MJ, Fan JH, et al. Follow-up of patients with multidrug resistant tuberculosis four years after standardized first-line drug treatment. PloS One. 2010;5: e10799. doi:10.1371/journal.pone.0010799

32. Lan NTN null, Lademarco MF, Binkin NJ, Tung LB, Quy HT, Cĵ NV. A case series: initial outcome of persons with multidrug-resistant tuberculosis after treatment with the WHO standard retreatment regimen in Ho Chi Minh City, Vietnam. Int J Tuberc Lung Dis Off J Int Union Tuberc Lung Dis. 2001;5: 575–578.

33. Grandjean L, Gilman RH, Martin L, Soto E, Castro B, Lopez S, et al. Transmission of Multidrug-Resistant and Drug-Susceptible Tuberculosis within Households: A Prospective Cohort Study. PLoS Med. 2015;12: e1001843. doi:10.1371/journal.pmed.1001843

34. Borrell S, Gagneux S. Infectiousness, reproductive fitness and evolution of drug-resistant Mycobacterium tuberculosis [State of the art]. Int J Tuberc Lung Dis. 2009;13: 1456–1466.
