# Supplementary material for: Priority-Setting for Novel Drug Regimens to Treat Tuberculosis: An Epidemiologic Model
Source: PLoS Med. 2017 Jan 3;14(1):e1002202. doi: 10.1371/journal.pmed.1002202 (PMC5207633; doi:10.1371/journal.pmed.1002202)
Supplement: S2 Table — (DOCX) [file pmed.1002202.s007.docx]

***Priority-setting for novel drug regimens to treat tuberculosis: An epidemiologic model***

**S2 Table: Calibration targets for all modeled epidemiologic settings***

|  | TB prevalence (/100k) | HIV-TB co-prevalence | Rifampin-resistant incident fraction |
| --- | --- | --- | --- |
| India (primary analysis) | 195 | 4% | 2.2% |
| Brazil | 52 | 17% | 1.4% |
| Philippines | 417 | 0.2% | 2% |
| South Africa | 696 | 61% | 1.8% |

* Reference: Global TB Report 2015, World Health Organization
