# Supplementary material for: Priority-Setting for Novel Drug Regimens to Treat Tuberculosis: An Epidemiologic Model
Source: PLoS Med. 2017 Jan 3;14(1):e1002202. doi: 10.1371/journal.pmed.1002202 (PMC5207633; doi:10.1371/journal.pmed.1002202)
Supplement: S3 Table — (DOCX) [file pmed.1002202.s008.docx]

***Priority-setting for novel drug regimens to treat tuberculosis: An epidemiologic model***

**S3 Table: Baseline TB incidence and mortality of calibrated models**

|  | **Baseline TB incidence, RS-TB regimen scenario (/100k/yr)** | | **Baseline TB mortality, RS-TB regimen scenario (/100k/yr)** | | **Baseline RR-TB incidence, RR-TB regimen scenario (/100k/yr)** | **Baseline RR-TB mortality, RR-TB regimen scenario (/100k/yr)** |
| --- | --- | --- | --- | --- | --- | --- |
|  | **Model result^(1)^** | **WHO estimate^(2)^** | **Model result^(3)^** | **WHO estimate^(3)^** | **Model result**^(4)^ | **Model result** |
| **India (primary analysis)** | 157  (113-187) | 167  (156-179) | 16 (9-23) | 17 (12-27) TB-only +  2.4 (2.0-2.9) HIV+TB | 6.0  (3.5-10.2) | 0.61  (0.33-1.14) |
| **Brazil** | 39  (9-55) | 44  (42-46) | 4.3  (1.0-7.1) | 2.6 (2.4-2.7) TB-only + 1.2 (0.9-1.6) HIV+TB | 1.0  (0.3-1.9) | 0.11  (0.03-0.23) |
| **Philippines** | 325  (231-401) | 288  (254-324) | 35  (18-50) | 10 (9.1-11) TB-only + 0.08 (0.06-0.11) HIV+TB | 11.0  6.3-19.2) | 1.1  (0.5-2.1) |
| **South Africa** | 737  (632-832) | 834  (737-936) | 116  (61-178) | 44 (41-48) TB-only + 134 (107-164) HIV+TB | 25.8  (15.9-42.6) | 3.9  (2.1-6.9) |

^(1)^ Model results are presented as median (95% uncertainty range) over all simulations

^(2)^ WHO estimates are point estimate and confidence interval from the World Health Organization’s Global Tuberculosis Report 2015.

^(3)^ WHO’s TB mortality estimates are separated into non-HIV (all of which are attributed to TB) and TB+HIV (an unknown fraction of which are attributable to TB).

^(4)^ WHO does not estimate RR-TB incidence or mortality directly.
