# Supplementary material for: Priority-Setting for Novel Drug Regimens to Treat Tuberculosis: An Epidemiologic Model
Source: PLoS Med. 2017 Jan 3;14(1):e1002202. doi: 10.1371/journal.pmed.1002202 (PMC5207633; doi:10.1371/journal.pmed.1002202)
Supplement: S4 Table — (DOCX) [file pmed.1002202.s009.docx]

***Priority-setting for novel drug regimens to treat tuberculosis: An epidemiologic model***

**S4 Table: Estimated 10-year mortality and incidence impact of optimal novel regimen for all modeled settings, relative to current-care projections**

|  | **TB incidence reduction, optimal RS-TB regimen** | **TB mortality reduction, optimal RS-TB regimen** | **RR-TB incidence reduction, optimal RR-TB regimen** | **RR-TB mortality reduction, optimal RR-TB regimen** |
| --- | --- | --- | --- | --- |
| **India** | 12.0% (6.5-21.9%) | 11.3% (5.8-20.4%) | 31.8% (17.6-46.4%) | 30.2% (17.6-43.7%) |
| **Brazil** | 10.6% (6.4-18.7%) | 9.4% (5.7-16.1%) | 29.3% (15.5-43.2%) | 28.4% (16.5-41.1%) |
| **Philippines** | 11.6% (6.6-23.0%) | 10.6% (5.3-21.2%) | 29.5% (16.0-44.5%) | 27.6% (15.4-42.6%) |
| **South Africa** | 9.7% (5.8-16.5%) | 9.3% (5.0-16.2%) | 30.1% (15.4-47.7%) | 30.3% (17.1-45.4%) |
