# Supplementary material for: Priority-Setting for Novel Drug Regimens to Treat Tuberculosis: An Epidemiologic Model
Source: PLoS Med. 2017 Jan 3;14(1):e1002202. doi: 10.1371/journal.pmed.1002202 (PMC5207633; doi:10.1371/journal.pmed.1002202)
Supplement: S5 Table — (DOCX) [file pmed.1002202.s010.docx]

***Priority-setting for novel drug regimens to treat tuberculosis: An epidemiologic model***

**S5 Table: Sensitivity analysis for non-equilibrium underlying RS-TB epidemic: Parameters used and resulting TB incidence**

|  | **Annual change in varied parameter, relative to its value at year 0** | **Average annual change in TB incidence, year 0 to year 10, with no novel regimen** |
| --- | --- | --- |
| **Equilibrium model** | NA | NA |
| **Declining transmission coefficient (β)** | -2% | -2.3% |
| **Declining rapid progression probability (ρ_-_)** | -2% | -2.6% |
| **Declining reactivation rate (α_-_)** | -4% | -2.2% |
| **Increasing TB diagnosis rate (χ_n-_)** | +5% | -2.2% |
