# Supplementary material for: Priority-Setting for Novel Drug Regimens to Treat Tuberculosis: An Epidemiologic Model
Source: PLoS Med. 2017 Jan 3;14(1):e1002202. doi: 10.1371/journal.pmed.1002202 (PMC5207633; doi:10.1371/journal.pmed.1002202)
Supplement: S6 Table — (DOCX) [file pmed.1002202.s011.docx]

***Priority-setting for novel drug regimens to treat tuberculosis: An epidemiologic model***

**S6 Table: Sensitivity analysis results for non-equilibrium epidemic: comparing impacts of improving a single regimen characteristic**

|  | **Fraction of total mortality impact achieved by optimizing single characteristic (median (95% UR))*** | | | | | |
| --- | --- | --- | --- | --- | --- | --- |
|  | **Efficacy** | **Barrier to resistance** | **Preexisting novel-regimen resistance** | **Medical Contraindications** | **Duration** | **Tolerability/ likelihood of adherence** |
| **Equilibrium model** | 44 (32-52)% | 21 (15-31)% | 0 (-4–3)% | -2 (-5–1)% | 23 (14-29)% | 17 (11-22)% |
| **Declining transmission coefficient (β)** | 43 (32-51)% | 23 (16-32)% | 0 (-4–2)% | -2 (-5–1)% | 23 (14-28)% | 17 (10-21)% |
| **Declining rapid progression probability (ρ_-_)** | 43 (32-51)% | 22 (16-31)% | 0 (-3—3)% | -2 (-5—1)% | 23 (14-28)% | 17 (10-21)% |
| **Declining reactivation rate (α_-_)** | 45 (33-53)% | 20 (15-31)% | 0 (-4—2)% | -2 (-5—1)% | 23 (15-30)% | 17 (11-22)% |
| **Increasing TB diagnosis rate (χ_n-_)** | 43 (32-52)% | 23 (17-32)% | 0 (-3—3)% | -2 (-5—1)% | 23 (14-29)% | 17 (10-22)% |

* Because these analyses were performed using a random 10% subset of the simulations used for the primary analyses, results for the equilibrium model differ slightly from those presented in the primary manuscript.
