# Supplementary material for: Priority-Setting for Novel Drug Regimens to Treat Tuberculosis: An Epidemiologic Model
Source: PLoS Med. 2017 Jan 3;14(1):e1002202. doi: 10.1371/journal.pmed.1002202 (PMC5207633; doi:10.1371/journal.pmed.1002202)
Supplement: S7 Table — (DOCX) [file pmed.1002202.s012.docx]

***Priority-setting for novel drug regimens to treat tuberculosis: An epidemiologic model***

**S7 Table: Sensitivity analysis results for non-equilibrium epidemic: comparing impacts of failing to optimize a single regimen characteristic**

|  | **Fraction of total mortality impact lost when single characteristic is not optimized (median (95% UR))*** | | | | | |
| --- | --- | --- | --- | --- | --- | --- |
|  | **Efficacy** | **Barrier to resistance** | **Preexisting novel-regimen resistance** | **Medical Contraindications** | **Duration** | **Tolerability/ likelihood of adherence** |
| **Equilibrium model** | 40 (33-54)% | 27 (21-39)% | 10 (7-13)% | 8 (6-10)% | 8 (5-13)% | 3 (2-4)% |
| **Declining transmission coefficient (β)** | 38 (32-51)% | 29 (22-40)% | 10 (8-13)% | 8 (6-10)% | 8 (5-12)% | 3 (2-4)% |
| **Declining rapid progression probability (ρ_-_)** | 38 (33-54)% | 29 (22-40)% | 10 (8-13)% | 8 (6-10)% | 8 (5-12)% | 3 (2-4)% |
| **Declining reactivation rate (α_-_)** | 39 (33-54)% | 27 (20-39)% | 10 (7-12)% | 8 (6-10)% | 8 (5-13)% | 3 (2-4)% |
| **Increasing TB diagnosis rate (χ_n-_)** | 38 (32-50)% | 30 (22-40)% | 10 (7-12)% | 8 (6-10)% | 8 (5-12)% | 3 (2-4)% |

* Because these analyses were performed using a random 10% subset of the simulations used for the primary analyses, results for the equilibrium model differ slightly from those presented in the primary manuscript.
