# Supplementary figures and images for: Priority-Setting for Novel Drug Regimens to Treat Tuberculosis: An Epidemiologic Model
Source: PLoS Med. 2017 Jan 3;14(1):e1002202. doi: 10.1371/journal.pmed.1002202 (PMC5207633; doi:10.1371/journal.pmed.1002202)

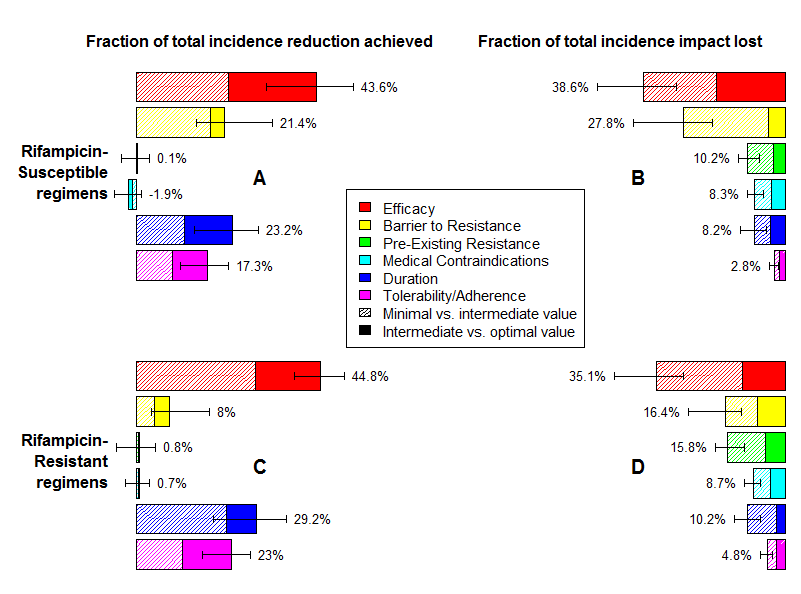

Supplement: S1 Fig — In contrast to other figures showing impact on the TB or RR TB mortality reduction resulting from a regimen, this analysis considers the impact of different novel regimen characteristics on the regimen’s ability to reduce TB incidence (RS TB regimens) or RR TB incidence (RR TB regimens). (TIF) [file pmed.1002202.s013.tif]

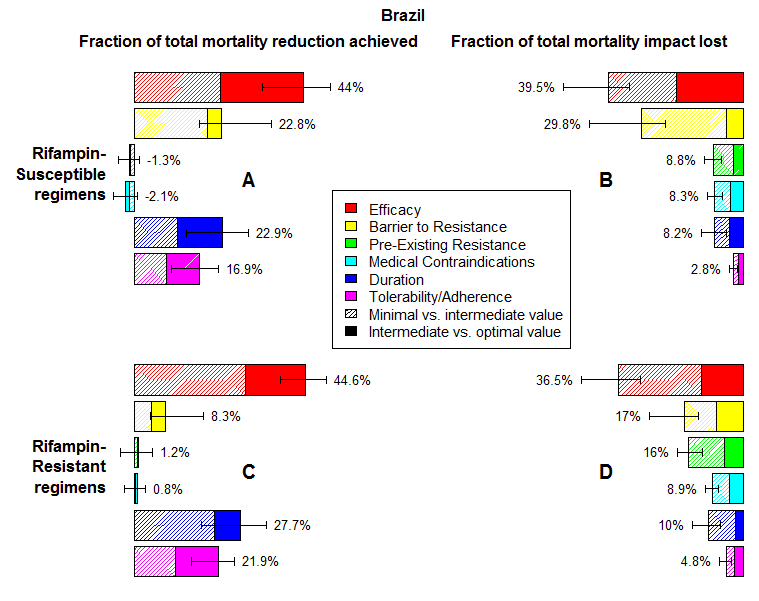

Supplement: S2 Fig — (TIF) [file pmed.1002202.s014.tif]

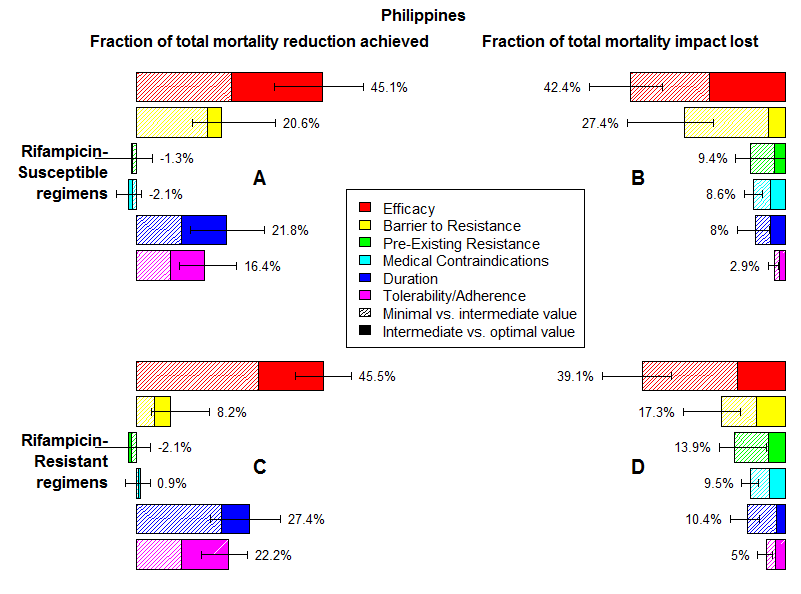

Supplement: S3 Fig — (TIF) [file pmed.1002202.s015.tif]

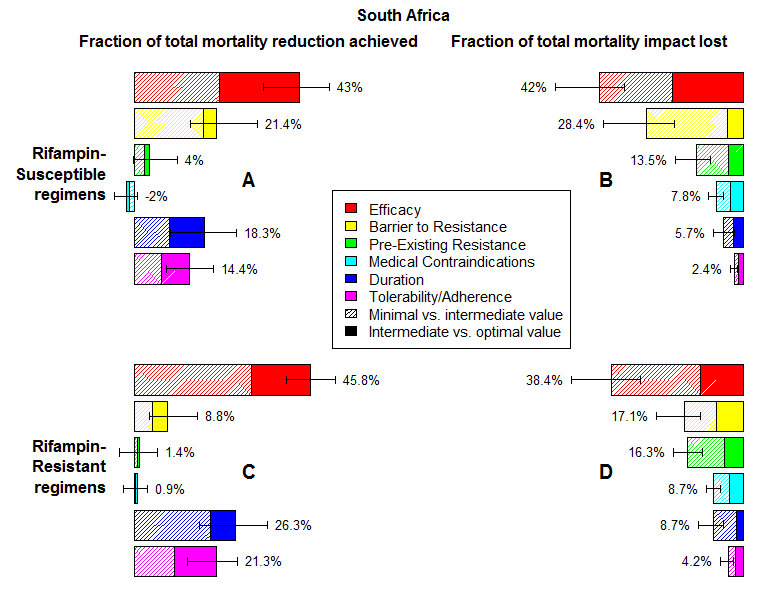

Supplement: S4 Fig — (TIF) [file pmed.1002202.s016.tif]

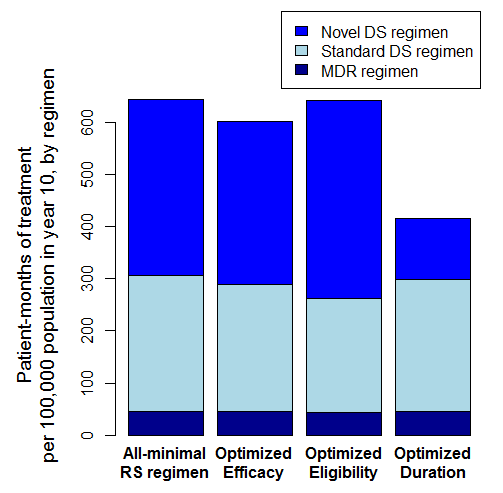

Supplement: S5 Fig — Different improvements in a novel RS TB regimen have different population-level impacts on total TB treatment person-time. Efficacy improvements reduce the use of all regimens by lowering incidence most dramatically. Inclusive eligibility allows more patients to receive the novel rather than standard regimen, which reduces total treatment time only if the novel regimen is also shorter. Shortening the regimen duration has a direct and immediate impact on the total patient-months on treatment. (TIF) [file pmed.1002202.s017.tif]

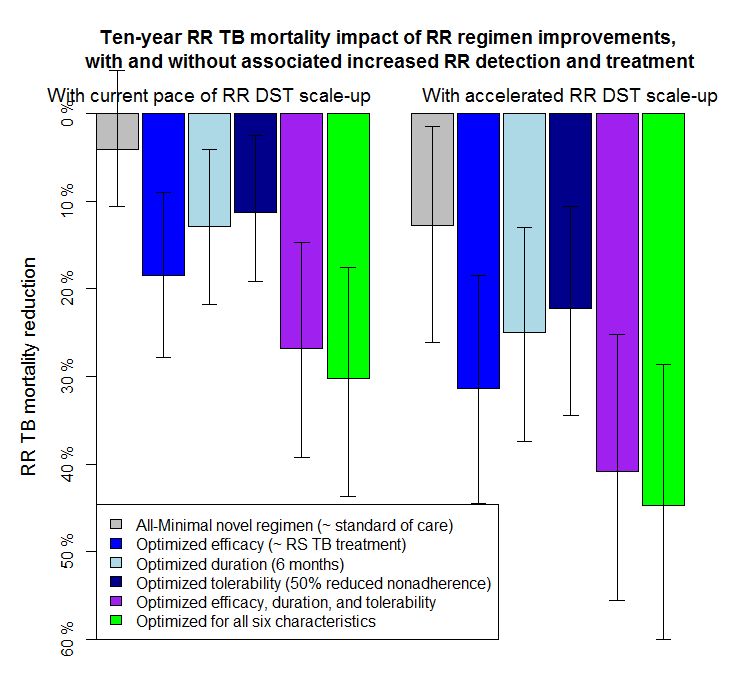

Supplement: S6 Fig — This sensitivity analysis considers a scenario in which an improved RR TB regimen allows or motivates more rapid scale-up of rifampin DST, such that universal rifampin DST is achieved by the end of the 3-y scale-up period for the novel regimen. Compared to the baseline scenario that assumes continued gradual scale-up of DST, the indirect effect of simultaneous rapid DST scale-up is expected to approximately double the direct effect of a novel regimen improvement such as shortened duration or improved tolerability. (TIF) [file pmed.1002202.s018.tif]

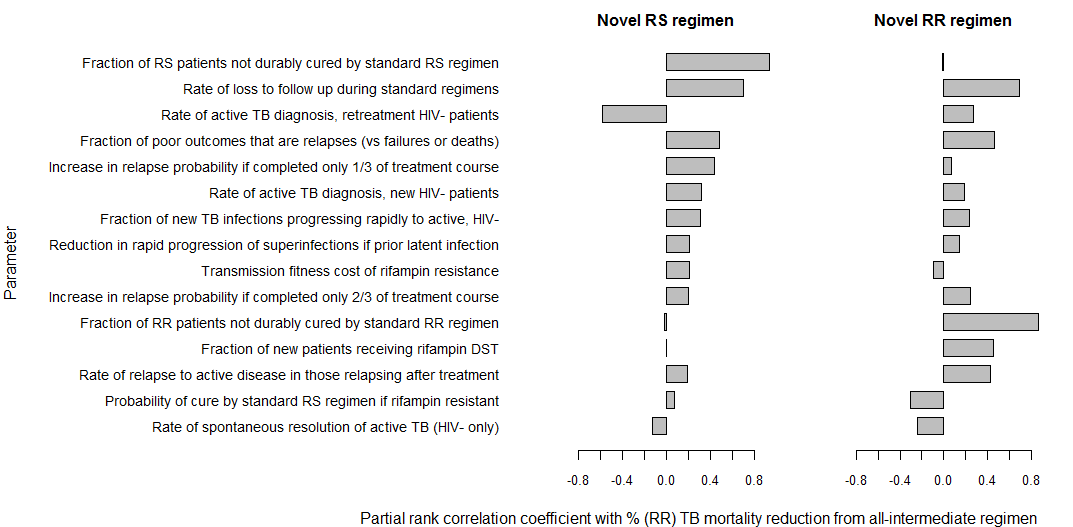

Supplement: S7 Fig — Partial rank correlation (adjusted for other parameters) was calculated for each parameter with the percent reduction in TB mortality (or, for RR TB regimens, the percent reduction in RR TB mortality) achieved by a novel regimen that met all intermediate target criteria. (TIF) [file pmed.1002202.s019.tif]

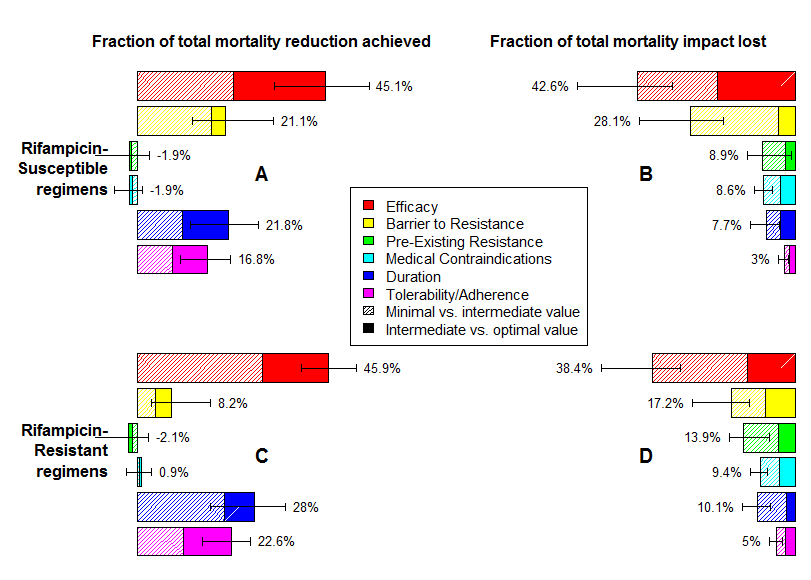

Supplement: S8 Fig — (TIF) [file pmed.1002202.s020.tif]

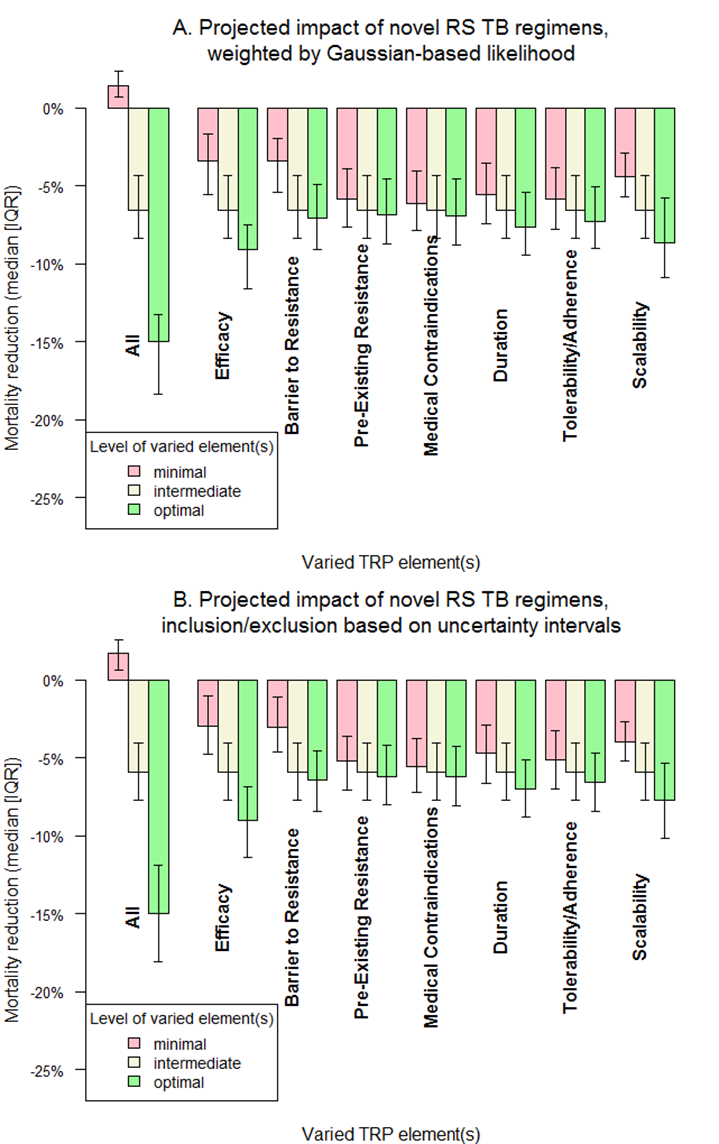

Supplement: S9 Fig — The calibration method used in the primary analysis, in which all simulations that fell inside of uncertainty intervals were included in the analysis with equal weight, is compared to an alternative approach weighted according to a Gaussian-based likelihood function as described in S3 Methods. In order to summarize many results in a single figure, regimen characteristics not being varied are set at an intermediate baseline; scalability is included among the characteristics varied; and reduction in mortality is shown relative to projections without any novel regimen. (TIF) [file pmed.1002202.s021.tif]
